# The Predatory Ecology of *Deinonychus* and the Origin of Flapping in Birds

**DOI:** 10.1371/journal.pone.0028964

**Published:** 2011-12-14

**Authors:** Denver W. Fowler, Elizabeth A. Freedman, John B. Scannella, Robert E. Kambic

**Affiliations:** 1 Museum of the Rockies and Department of Earth Sciences, Montana State University, Bozeman, Montana, United States of America; 2 Department of Ecology and Evolutionary Biology, Brown University, Providence, Rhode Island, United States of Ameria; Raymond M. Alf Museum of Paleontology, United States of America

## Abstract

Most non-avian theropod dinosaurs are characterized by fearsome serrated teeth and sharp recurved claws. Interpretation of theropod predatory ecology is typically based on functional morphological analysis of these and other physical features. The notorious hypertrophied ‘killing claw’ on pedal digit (D) II of the maniraptoran theropod *Deinonychus* (Paraves: Dromaeosauridae) is hypothesized to have been a predatory adaptation for slashing or climbing, leading to the suggestion that *Deinonychus* and other dromaeosaurids were cursorial predators specialized for actively attacking and killing prey several times larger than themselves. However, this hypothesis is problematic as extant animals that possess similarly hypertrophied claws do not use them to slash or climb up prey. Here we offer an alternative interpretation: that the hypertrophied D-II claw of dromaeosaurids was functionally analogous to the enlarged talon also found on D-II of extant Accipitridae (hawks and eagles; one family of the birds commonly known as “raptors”). Here, the talon is used to maintain grip on prey of subequal body size to the predator, while the victim is pinned down by the body weight of the raptor and dismembered by the beak. The foot of *Deinonychus* exhibits morphology consistent with a grasping function, supportive of the prey immobilisation behavior model. Opposite morphological trends within Deinonychosauria (Dromaeosauridae + Troodontidae) are indicative of ecological separation. Placed in context of avian evolution, the grasping foot of *Deinonychus* and other terrestrial predatory paravians is hypothesized to have been an exaptation for the grasping foot of arboreal perching birds. Here we also describe “stability flapping”, a novel behaviour executed for positioning and stability during the initial stages of prey immobilisation, which may have been pivotal to the evolution of the flapping stroke. These findings overhaul our perception of predatory dinosaurs and highlight the role of exaptation in the evolution of novel structures and behaviours.

## Introduction

From its description by John Ostrom in 1969 [1], the Early Cretaceous carnivorous dinosaur *Deinonychus antirrhopus* (Theropoda: Dromaeosauridae) became an icon of the “dinosaur renaissance”. Depictions of *Deinonychus* as a fleet, intelligent predator, operating in packs to take down prey much larger than itself [2]–[4], captured the imagination of the public and researchers alike. Interest has grown yet further in recent years, following cladistic analyses that recovered Deinonychosauria [5] (Dromaeosauridae + Troodontidae) as the sister group to birds, prompting debate as to how flight might have evolved from a deinonychosaurian-like ancestor. Despite this level of interest, much of what is typically assumed about the ecology of *Deinonychus,* and Deinonychosauria in general, is based on speculation. Although the enlarged pedal D-II claw has generated much interest, surprisingly few analyses have compared dinosaur claw morphology to animals with known ecologies [6], [7], mainly because of a paucity of research on claw morphology and function in general ([4], [7], [8]; although see [9], [10]). In a novel experiment, Manning et al. [2] demonstrated that the hypertrophied D-II claw would not be effective for slashing and suggested instead that it was used by dromaeosaurids as a climbing crampon for gripping the hides of prey several times larger than themselves (see Supporting Information Text S1 for further review). We agree that the D-II claw is most effective as a hooked device, but modern analogues that have similarly hypertrophied D-II claws do not use them to climb up prey.

In extant birds, variation in foot morphology is associated with variation in behaviour and factors such as speed, strength, agility, even diet [6], [8], [11]–[21]. Our recently published sibling study [20] investigated how foot morphology is related to predatory behavior in extant birds of prey. We showed for the first time that the Accipitridae (hawks and eagles) also possess a conspicuously hypertrophied talon on D-II and that this is utilized for prey immobilisation.

It is important for extant predators to quickly subdue their victims, lest they escape or retaliate against their attacker. In extant raptors, prey immobilisation strategy is variable and mostly dependent on relative prey size [20] (“immobilisation” is preferred to “killing” because accipitrids often do not wait until the death of their victims before feeding [22], [23]). In all birds of prey, small prey (those that can be contained within the foot [20]) are immobilized by containment within the foot, assisted by constriction and beak attacks [22]–[26]. Physical adaptations for increasing foot strength (hence constriction ability) are more developed in owls which are small prey specialists [19]. In falcons and some owls, immobilisation is aided by attempts to snap the spinal cord or crush the head with the predator's beak. Falcons have evolved a stronger bite force and a specialized “tomial tooth” on the beak to aid in doing so [27], [28]. Large prey are defined as being too big to be contained within the foot, and so cannot be constricted [20] (Supporting Information Videos S1 and S2). To prevent escape of large prey the raptor pins its victim to the ground using its bodyweight, then plucks away feathers or fur, exposing an area of flesh. For immobilisation, falcons will quickly attempt to snap the spinal cord to kill the prey, but accipitrids lack the physical specializations for this. Instead, accipitrids possess hypertrophied talons on D-I and D-II which are adaptations for maintaining grip on large struggling prey [20]. Accipitrids' talons lock into their prey, keeping hold despite vigorous struggling, allowing the raptor to begin feeding. In such cases, death of the victim is hastened by massive bleeding from wounds sustained whilst being eaten alive.

**Figure 1:**
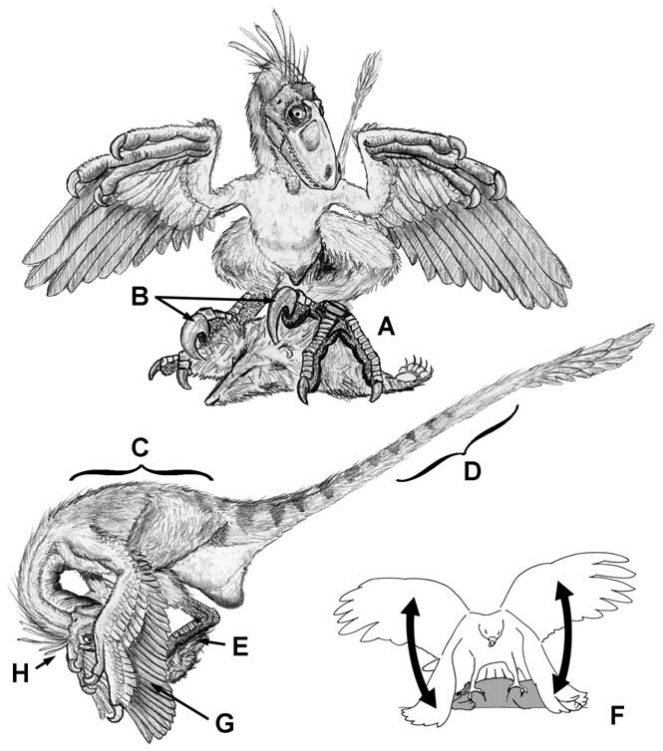
RPR “ripper” behavioural model, illustrated by a small dromaeosaurid. (**A**) grasping foot holds on to prey. (**B**) hypertrophied D-II claw used as anchor to maintain grip on large prey. (**C**) predator's bodyweight pins down victim. (**D**) beam-like tail aids balance. (**E**) low-carried metatarsus helps restrain victim. (**F**) “stability flapping” used to maintain position on top of prey (see Supporting Information Videos S1 and S2). (**G**) arms encircle prey (“mantling”), restricting escape route. (**H**) head reaches down between feet, tearing off strips of flesh (may explain unusual deinonychosaurian dental morphology). Victim is eaten alive or dies of organ failure.

**Figure 2:**
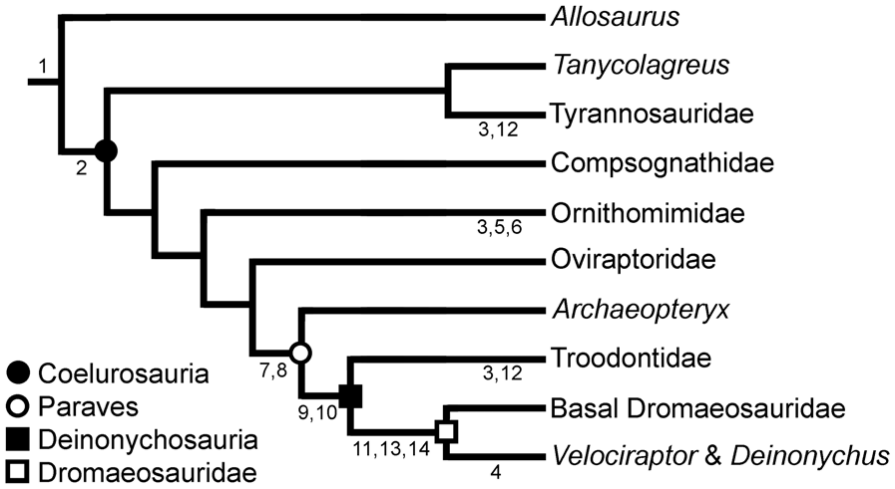
Phylogenetic distribution of characters pertinent to the RPR model. **1**. D-II ungual larger than D-III; **2**. elongate metatarsus; **3**. arctometatarsalian metatarsus; **4**. short robust metatarsus; **5**. dorso-ventrally flattened pedal unguals; **6**. D-II ungual smaller than D-III; **7**. elongate D-IV; **8**. hyperextensible D-II; **9**. enlarged D-II ungual; **10**. subarctometatarsalian metatarsus; **11**. hypertrophied D-II ungual; **12**. reduced forelimbs; **13**. stiffened tail; **14**. ginglymoid distal articulations of metatarsals. Phylogeny from Senter [5].

An understanding of how foot morphology affects predatory ability in extant birds of prey can inform interpretations of similar variation observed in extinct non-avian theropods. Previous comparisons of dinosaur hindlimb morphology have mainly concentrated on its contribution to locomotion ([29] and references therein). However, the hindlimb is more than just a component of the locomotor system, and in many theropod taxa the hindlimb exhibits features consistent with hooking and grasping functions. The purpose of this paper is to elucidate functional morphology of the deinonychosaurian pes by comparison to the findings of our sibling study [20], referred to hereafter as the Raptor Prey Restraint (RPR, or “ripper”) model (Figure 1). When this approach is combined with consideration of phylogenetic trends already recognized within theropoda (Figure 2), many of the peculiarities of deinonychosaurian anatomy can be interpreted as adaptations associated with specific predatory behaviours. We suggest the enlarged D-II claws of deinonychosaurians were used to grapple prey in a fashion comparable to accipitrid birds of prey, and are part of a suite of features that indicate ecological separation within Deinonychosauria and Paraves. These findings open several new lines of research into the predatory abilities of extinct theropods, and the evolution of novel structures and behaviours leading to birds. This includes description of a new flapping behaviour that may have important implications for the origin of flight.

## Methods

This paper is a qualitative application of the RPR model to non-avian theropods which is based on a quantitative assessment of predatory morphology and behaviour in birds of prey [20]. Measurements were taken of non-avian theropod specimens to test predictions of the RPR model and to emphasize trends in hindlimb proportions described elsewhere [15]. Through our work on birds of prey, it is clear that consideration of each digit is essential, as the way in which each varies is an indication of predatory specialty or locomotor habit. Previous analyses have considered only one digit (typically D-III [6]-[8], [15]), and are much less able to assess variation in foot use. Indeed, proportions of accessory digits D-II and D-IV vary more strongly than D-III, depending on use.

Principal morphological observations of Dromaeosauridae were made on MOR 747 (two complete and one partial pes of *Deinonychus antirrhopus*, Cloverly Formation, Aptian-Albian, Montana; Museum of the Rockies, Bozeman, MT, USA). Examination of well-preserved troodontid pedal material (MOR 553S.TM068 and MOR 748.TM065: *Troodon* sp., Two Medicine Formation, Campanian, Montana) permitted further comparison within Deinonychosauria.

Measurements were recorded from 52 non-avian theropod specimens (26 taxa, MNI = 45) and (where appropriate) added to the extant raptor dataset [20]. Where possible we measured metatarsus length, ungual size and curvature, and non-ungual phalanx length for all pedal digits. Some non-ungual measurements were taken directly from published descriptions. Claw attributes and measurements follow the nomenclature of Fowler et al. [20]. Statistical analysis (correspondence analysis, one-tailed t-tests assuming equal variances) was conducted on the combined dataset. Correspondence analyses (CA) were run in the R language and environment for statistical computing (version 2.11.1 for Mac OSX: www.R-project.org; [30]), to determine whether taxa group by pes morphology. Correspondence analyses were used because they are less susceptible than principal components analysis to outliers. Phalangeal and ungual measurements were converted into ratios to remove the effects of body size. In the first CA, two complete pedes of *Deinonychus* specimen MOR 747 were measured and added to the complete dataset of Fowler et al. ([20] Supporting Information; n = 42). The second CA focused on phalangeal proportions of non-avian dinosaurs, and excluded some incomplete specimens from the dataset (n = 30). Original data can be found in Supporting Information Table S1.

Although Ostrom [1] considered the range of motion for the D-II ungual and penultimate non-ungual phalanges of *Deinonychus*, he only briefly mentioned the range of motion for the other digits. In order to ascertain range of motion, we manually manipulated 2 partial, and one complete pes, (MOR 747; following the method of Senter [31], [32]). Metatarsal (MT)-I was reconstructed in an unreversed position to match that seen in articulated specimens of *Velociraptor mongoliensis* ([33], [34], *contra* Ostrom [1]).

Phylogenetic nomenclature follows that of Senter [5], except that we use the term “Ornithomimidae” in place of his “Arctometatarsalia”. The latter term is potentially confusing, since ornithomimids, tyrannosaurids, troodontids and some other theropods all exhibit the arctometatarsalian condition of the pes (and this is something we discuss frequently in this paper), yet Senter's use of the term refers to what many workers would understand as Ornithomimidae only, hence our change. Phalangeal nomenclature follows Moreno et al. [35] where appropriate.

Photographs were taken using Canon S400 and SX110 cameras. Figures were processed using Adobe Photoshop.

## Results

Most non-avian theropods measured possess a slightly more curved and enlarged ungual on D-II: in non-paravian theropods it is ∼10% larger than the ungual of D-III (e.g. D-II is 14.5% larger than D-III in *Tyrannosaurus*, 11.3% larger in *Allosaurus*). The D-II ungual is enlarged in Deinonychosauria relative to non-paravian dinosaurs (one-tailed t-test assuming equal variances, alpha  = 0.05, n = 10: D-II/D-III t_8_ = 3.471, p = 0.004); however, it is only especially hypertrophied in derived dromaeosaurids, where it is associated with further shortening of the non-ungual phalanges of D-II.

### Correspondence analyses

**Figure 3:**
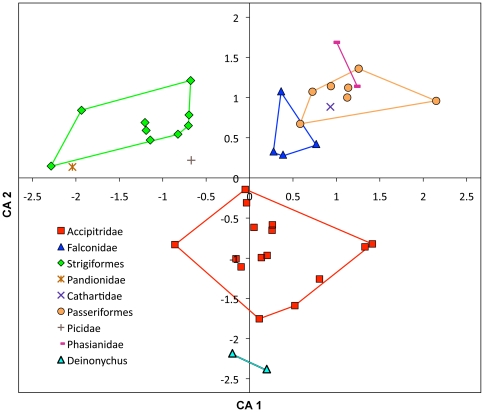
Correspondence Analysis comparing relative ungual and digit sizes of *Deinonychus* and extant avians. *Deinonychus* plots nearest to Accipitridae, emphasizing similarity in pedal morphology. Axis 1  = 50.68% of variation, Axis 2 = 24.15% of variation. Extant avian data (mostly birds of prey) from Fowler et al. [20]. n = 42.

When *Deinonychus* is plotted in a CA with extant birds of prey, it falls closest to the accipitrids, although even more negative along axis 2. This morphological similarity in pes dimensions is driven by their enlarged D-II unguals and relative proportions of other unguals (Figure 3).

**Figure 4:**
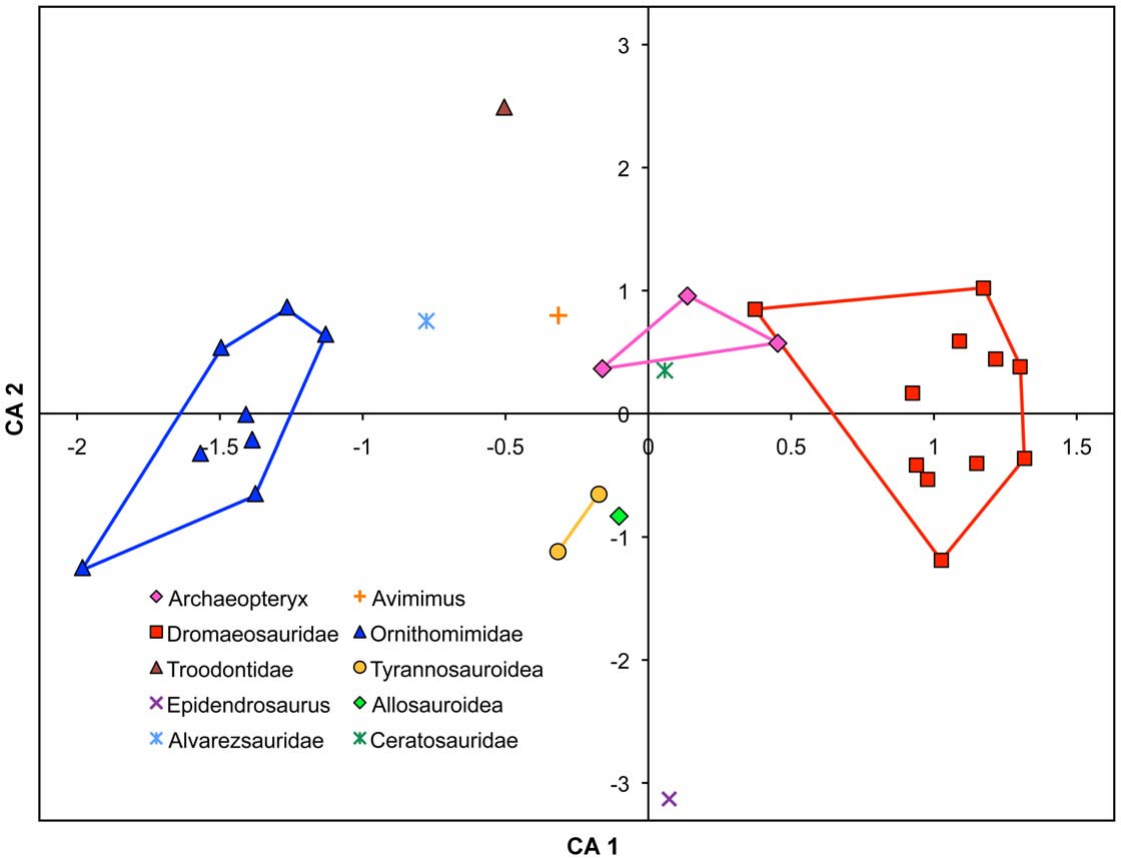
Correspondence Analysis comparing relative proportions of pedal non-ungual phalanges among non-avian theropod dinosaurs. Separation along Axis 1 (64.99% of variation) discriminates cursorial Ornithomimidae from less-cursorial Dromaeosauridae. Troodontidae plot closer to Ornithomimidae than their sister-taxon Dromaeosauridae, indicating a more cursorial habit. *Archaeopteryx* plots close to Dromaeosauridae, but in a more intermediate position, as do Tyrannosauroidea, Allosauroidea, and Ceratosauridae. The separation of *Archaeopteryx* from Tyrannosauroidea and Allosauroidea along Axis 2 (13.89% of variation) suggests an additional discriminatory aspect of phalanx proportions. n = 30.

The CA of non-avian dinosaur phalangeal proportions clusters specimens by taxonomic group and lifestyle (Figure 4). The first axis, which explains 64.99% of the variation, separates the strongly cursorial Ornithomimidae from other theropods. Intermediate taxa include the less strongly cursorial tyrannosauroids, *Archaeopteryx*, *Dilophosaurus*, *Avimimus*, *Troodon*, and *Epidendrosaurus*. These are spread widely along the second axis, which explains 13.89% of the variation. On the negative side of Axis 2, the tyrannosauroids cluster together, and the unusual *Epidendrosaurus* appears to possess exaggerated tyrannosauroid phalangeal proportions. On the positive side of Axis 2, *Avimimus* falls amongst the *Archaeopteryx* specimens, with *Troodon* farther along the axis. *Troodon* and *Epidendrosaurus* have similar eigenvalues along Axis 1, yet are the most widely separated taxa along Axis 2. While the CA analyses show some grouping, assessment of ratios individually is more informative (see discussion), partly because terrestrial grasping predators must exhibit a compromise of cursorial vs grasping characters.

### Biomechanical analysis MOR 747 (*Deinonychus*)

The distal articular surface of MT-I has a distinct twist, rotating phalanx D-I-1 to face more laterally, although there appears to be little possible movement at this joint, restricting D-I-1 to a relatively fixed position. The distal articular facet of D-I-1 is ginglymoid, restricting the ungual (D-I-2) to vertical motion.

**Figure 5:**
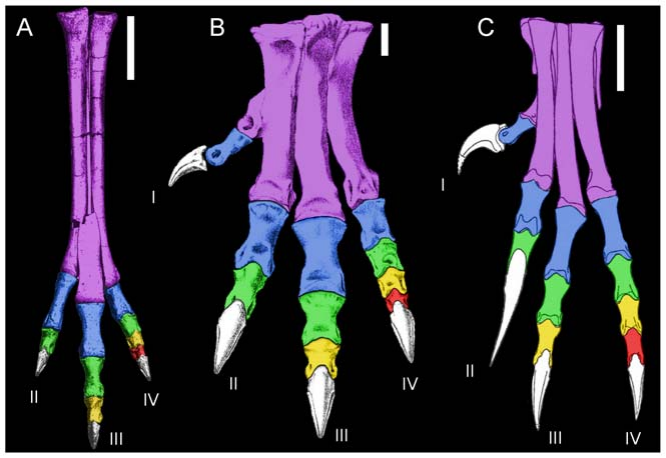
Variation in foot proportions consistent with cursoriality or grasping. Cursorial-proportioned feet of *Gallimimus* (**A**) and *Allosaurus* (**B**) exhibit D-II and D-IV of subequal lengths, with D-IV significantly shorter than D-III. This is contrasted with *Deinonychus* (**C**) where D-IV is significantly elongated, being subequal in length to D-III, with distal-most non-ungual phalanges of D-III and IV subequal in length to the preceding penultimate non-ungual phalanx; features consistent with a grasping habit [20]. Scale  = 5 cm. Modified from original sources [1], [42], [100].

The shaft of MT-II is mostly straight but exhibits a variable amount of medial deflection in the distal third. The shaft of MT-III is straight. The distal articular surfaces of MT-II and MT-III, and those of individual phalanges in each digit, are ginglymoid, limiting these joints to movement in a single dorso-ventral plane. The distal articular facets of MT-II and MT-III show slight medial deflection, so that *contra* the reconstruction of Ostrom ([1]: Figure 73) it is not physically possible for the phalanges of D-III to articulate in a straight line parallel to the shaft of MT-III without disarticulating MT-III and D-III-1. Instead, D-II and D-III are typical of non-arctometatarsalian theropods in that both are oriented slightly medially with respect to the metatarsus, and very slightly divergent with respect to each other (illustrated in Figure 5). The shaft of MT-IV is straight in the proximal two thirds, but deflects laterally in its distal third. MT-IV has a ball-like distal articular facet, matched by a concave proximal articular facet of phalanx D-IV-1 allowing D-IV some variability in lateral positioning. D-IV-1 has slight medial curvature over its length, deflecting D-IV towards D-III.

During extension, D-IV articulates in a relatively restricted manner, placing the toe as slightly divergent from D-III. D-II and D-III are roughly parallel as the distal end of MT-III is deflected medially (Figure 5; *contra* Ostrom [1]). This is important as it alters the interdigital divarification angle, which might be used to interpret didactyl footprints as either troodontid or dromaeosaurid.

**Figure 6:**
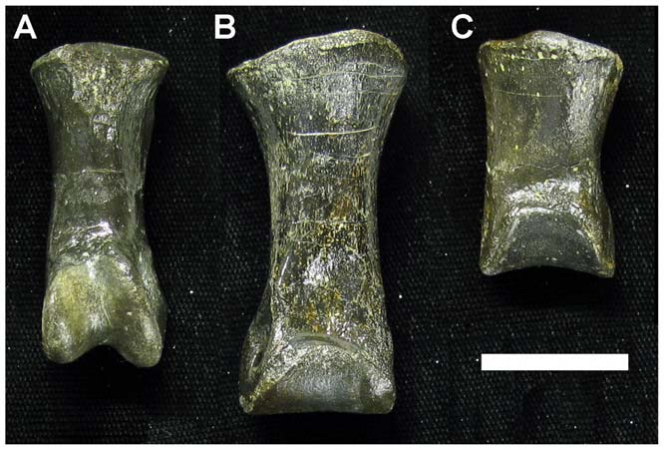
Comparison of ginglymoid vs non-ginglymoid articulation facets in first pedal phalanges of *Troodon* sp. (all dorsal view). The distal articulation facet is ginglymoid in D-II-1 (**A**; MOR 553S-6.29.9.89), but not in D-III-1 (**B**; MOR 553S-8.11.9.209) or D-IV-1 (**C**; MOR 553S-8.11.92.213). Specimens are derived from a multi-individual bonebed and may not be from the same individual, hence differences in size are not relevant. Scale bar  = 2 cm.

**Figure 7:**
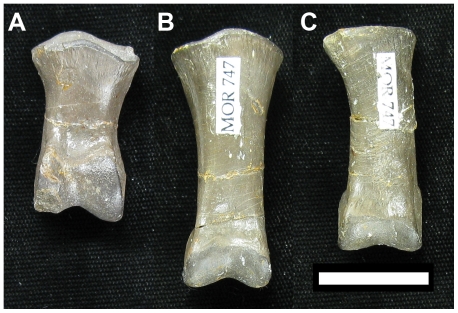
Comparison of ginglymoid vs non-ginglymoid articulation facets in first pedal phalanges of *Deinonychus* (MOR 747; all dorsal view). The distal articulation facet is ginglymoid in D-II-1 (**A**), D-III-1 (**B**) and more weakly so in D-IV-1 (**C**). Specimens found as part of an articulated pes. Scale bar  = 2 cm.

In *Troodon* sp. (MOR 553S.TM068 and MOR 748.TM065) the distal articular ends of the metatarsals are not ginglymoid, and the only pedal phalanges that exhibit ginglymoid articulation facets are all phalanges of D-II, and the distalmost non-ungual phalanges of D-I, D-III and D-IV (Figure 6; as seen in other troodontids. [36]–[38]. All other phalanges are non-ginglymoid. By comparison, in basal dromaeosaurids the distal articular ends of the metatarsals are either non-ginglymoid or weakly ginglymoid [39]–[41], developing into strongly ginglymoid in derived forms (MOR 747). In dromaeosaurids interphalangeal articulation facets are usually ginglymoid on all digits ([1]; Figure 7).

## Discussion

### The RPR (“ripper”) model

Under the RPR model (Figure 1), the grasping foot of Deinonychosauria is interpreted as an adaptation for holding on to prey as the predator's bodyweight pins down its victim. Positioning and balance is maintained by anchoring the hypertrophied D-II claw into the prey (also preventing escape), assisted by “stability flapping” (see supplementary videos) and movement of the beam-like tail. Prey escape is restricted as the forelimbs encircle prey (“mantling”). Deinonychosaurians lack any obvious specializations for prey dispatch, and so were probably similar to accipitrids in eating their prey alive. Detailed discussion of the morphological evidence behind these various components of the RPR model is presented below.

### Foot proportions and functional interpretation

Foot proportions vary considerably among clades of non-avian theropod dinosaurs (Figures 2 and 5). Although combinations of foot morphological characters are not identical to extant raptors (i.e. we do not see one taxon as an “owl mimic” another as a “falcon”, etc.) likely predatory behaviours can be elucidated by comparison of individual characters and their functional morphology in extant birds of prey. A detailed investigation into all extinct non-avian theropods is beyond the scope of this manuscript, but some initial findings are worthy of reporting, helping us to understand the origin and development of RPR model behaviour (Figure 1).

### D-II claws and pinning behaviour

The large size and high curvature of the D-II claw in Deinonychosauria is suggestive of its use in pinning down prey, as is its function in extant Accipitridae. Positioned on the inside of the foot and on a relatively short toe, the D-II ungual is best served to exert maximum leverage. In extant carnivorous birds (including non-predaceous forms such as crows and turkey vultures), D-II bears the largest claw (of D-II to IV) and is used for pinning down of food items during consumption [20]. Actively predaceous carnivores (such as Accipitridae and Falconidae) typically exhibit greater curvature of the D-II ungual than taxa that are only scavengers ([20]; although see Supporting Information Text S1). In most extinct carnivorous non-avian theropods, D-II also bears the largest ungual, and we suggest it was used for a similar pinning function. By contrast, in non-carnivorous extant birds (which do not exhibit pinning behaviours) the D-III claw is the largest, and the claws are generally less curved than carnivorous taxa [6], [20]. Similarly, in the secondarily herbivorous theropod dinosaurs Ornithomimidae (Figure 5; [42]) and *Avimimus* [43], the ungual of D-III is the largest, and all unguals have relatively very low curvature (consistent with a cursorial habit for these taxa). Further, ornithomimids and *Avimimus* exhibit reduction in size of pes flexor tubercles, which suggests considerably reduced strength in the foot. These are all features expected of non-carnivores that do not use the D-II ungual (or indeed any unguals) in pinning or any other predatory behaviour. Thus, curvature and / or relative size of the D-II ungual potentially provides independent indication of carnivory, or active predation [9], which may have implications for recently proposed hypotheses of herbivory in coelurosaurian dinosaurs ([44]; see discussion in Supporting Information Text S1). Pinning of prey by more basal non-avian theropods may have served as a behavioural origin for the RPR model in Paraves (Avialae + Troodontidae + Dromaeosauridae; *sensu* Senter [5]) where prey manipulation by the feet is hypothesized to have become increasingly important.

### Cursorial and grasping feet

Relative proportions of the feet vary depending on a predominantly cursorial or grasping function (Figure 5). Extant cursorial birds (e.g. ratites; Emu, *Dromaius novaehollandiae*, MOR OST-1299) and similar cursorially adapted dinosaur taxa (e.g. Ornithomimidae [42]; *Avimimus* [43]) typically exhibit a robust D-III, foreshortened distal phalanges with shorter, subequally sized lateral digits (D-II and IV) [11], [13]–[15], [17], [45]–[48]. An opposite trend is seen in the pedal digits of Deinonychosauria and basal Avialae (e.g. *Archaeopteryx*): D-IV becomes especially elongated, distal non-ungual phalanges are more elongated than in specialist cursors, and D-II becomes hyperextensible. These characters are consistent with grasping rather than a cursorial function [15].

Elongation of the metatarsus increases length of the flexor tendons, thus reducing mechanical advantage (and hence, grip strength), but also increases stride length (with other cursorial benefits [46], [48]) and ‘quickness’ of the feet. Extant accipitrids have an elongate metatarsus compared to other birds of prey, granting them better ability to snatch prey, an important aspect of their predatory strategy [19], [20]. Conversely, acting as the out-lever of the hindlimb, the short robust metatarsus of owls effectively reduces the length of the flexor tendons, increasing mechanical advantage; hence owls have greater force production (grip strength) than that produced by the more elongate metatarsus of accipitrids, but at a cost of less rapid movement [19]. A relatively elongate metatarsus (sometimes subarctometatarsalian) is present in basal paravians (e.g. basal troodontid *Sinovenator* [49]; basal dromaeosaurid *Sinornithosaurus* [50]; [51], [52]), indicating a cursorial habit is basal for the clade. This is further developed into a fully arctometatarsalian metatarsus in derived troodontids [5], [37], [38], [46], [53], [54]. By contrast, derived dromaeosaurids like *Deinonychus* [1], *Saurornitholestes* (MOR 660), and *Velociraptor* [33] lost the elongate metatarsus, instead evolving extremely short and robust metatarsi (among the shortest relative to tibia length of all non-avian theropods [55]). This suggests that the plesiomorphic cursorial metatarsus became further adapted towards cursoriality in Troodontidae whereas Dromaeosauridae reversed selection direction, specializing towards grasping strength at the expense of speed (Figure 2).

The morphology of interphalangeal articulation surfaces is indicative of strategy for countering stress incurred during foot use. Ginglymoid phalangeal articulations limit movement of the joint to a single plane, affording resistance to torsion but decreasing flexibility [35]. Conversely, non- or weakly ginglymoid articulations (“roller” joints) are indicative of low torsional stresses, and are especially prevalent in cursorial taxa on digits that are mediolaterally oriented relative to the ground resistant force [35]. In the cursorial ratites (e.g. emu, MOR-OST 1299) and similarly cursorial ornithomimids (e.g. *Ornithomimus* sp. UCMP 154569), the main weight of the animal is borne on the central digit (D-III; [35], [56], [57]) the phalanges of which are nearly symmetrical in shape and exhibit a very weak sagittal furrow (i.e. non-ginglymoid). The lateral digits (D-IV and D-II, where present) perform a stabilization role [35], [56] and exhibit ginglymoid interphalangeal articulations (except for ungual articulations, which are non- to weakly ginglymoid on all digits). In extant birds of prey, all interphalangeal articulations are strongly ginglymoid, which might be expected as struggling prey exact torsional loads on the predator's feet. In basal dromaeosaurids, the distal articular ends of the metatarsals are either non-ginglymoid or weakly ginglymoid [41], [50], [52]. In derived dromaeosaurids, all metatarsal and interphalangeal joints are ginglymoid ([1], except for MT-IV; see above), indicating adaptation for torsional resistance. By contrast, in the derived troodontid *Troodon* sp. (MOR 553S.TM068; also seen in other troodontids; [36]–[38]), the only ginglymoid joints are interphalangeal articulations of D-II and all ungual articulations. D-III bears roller joints, and D-IV is only weakly ginglymoid (Figure 6). This suggests a more strongly cursorial habit than seen in Dromaeosauridae (Figure 7), but somewhat different from the cursorial style of extant ratites, perhaps due to the didactyl pes of troodontids. Also, it is interesting that the unguals of MOR 553S.TM068 maintain ginglymoid articulations, even when other non-ungual interphalangeal articulations in the same digit do not. This is unlike that observed in ratites and ornithomimids, and may be explained as the unguals are probably still utilized for prey manipulation in troodontids, and so require some resistance to torsion.

**Figure 8:**
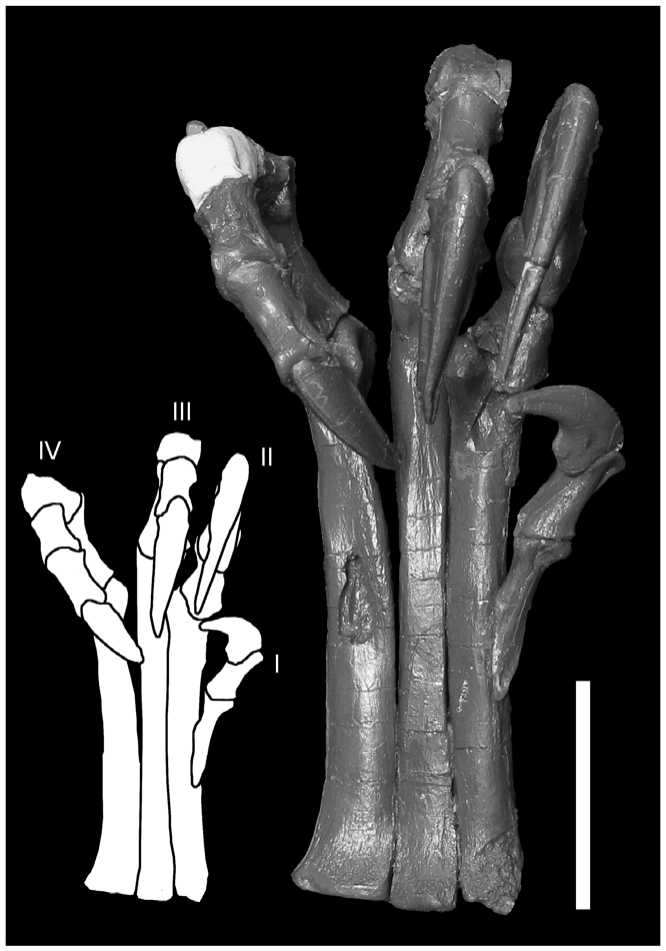
Ventral view of *Deinonychus* foot (MOR 747) in flexion. D-I is not reversed, but is rotated slightly so that the claw faces laterally into the ‘fist’, as observed in articulated specimens of *Velociraptor* [22]. Ginglymoid articulation facets of MT-II and III restrict the motion of D-II and III to a parallel dorso-ventral plane, but the distal ball joint of MT-IV allows D-IV to take a variable position, spreading more laterally, or allowing it to reach over the metatarsus, opposing D-I. Not shown at maximum flexion. Scale  = 5 cm.

A grasping interpretation for the pes of dromaeosaurids is further supported by analysis of range of motion in *Deinonychus*, as the pes forms an enclosed fist even without engaging maximum flexion (Figure 8). In contrast to the condition seen in most modern birds, where a fully reversed D-I opposes D-III (anisodactyly), in *Deinonychus* a medially directed D-I opposes D-IV, with D-II and III moving in parallel (*contra* Ostrom [1]), enclosing the fist antero-posteriorly across the “palm” of the metatarsus (aided by the low angle with which the metatarsus is carried relative to the substrate [58]). This arrangement is somewhat similar to when owls and ospreys rotate D-IV into a partially zygodactyl arrangement, thought to provide a more complete or even grasp. Similarity of the *Deinonychus* pes to other dromaeosaurids suggests that the grasping foot is typical of Dromaeosauridae as a whole (independently confirmed by Senter [32], and comparable phalangeal measurements among dromaeosaurid taxa; Supporting Information Table S1). Although many theropods possess a medially directed D-I and the ability to flex the foot tightly [32], only paravians possess the extreme digital elongation facilitating greater opposability and hence grasping ability.

**Figure 9:**
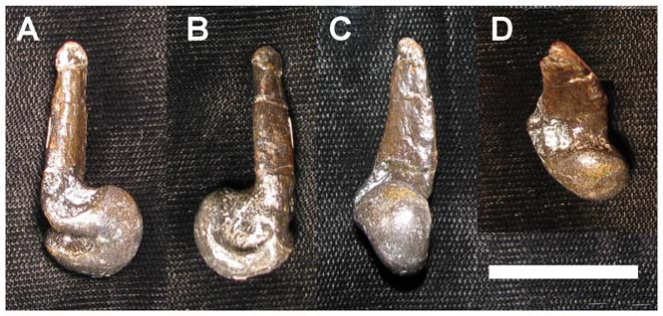
MOR 553S-8.6.92.168, *Troodon* sp. left MT-I in posterior (A), anterior (B), medial (C), and dorsal (D) views. MT-I has a ball-shaped articulation facet, allowing greater movement and positioning of D-I compared to MT-I of *Deinonychus* (Figure 10). Scale bar  = 2 cm.

**Figure 10:**
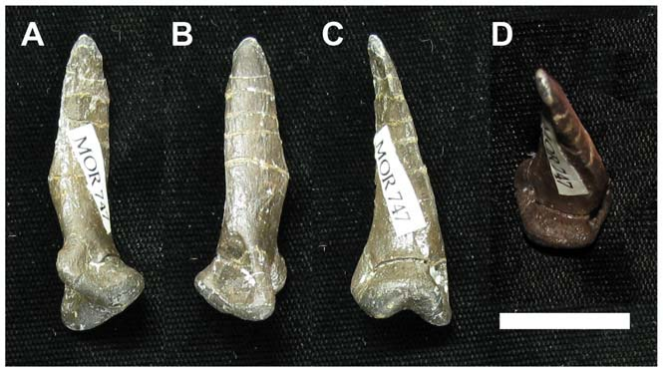
MOR 747, *Deinonychus* left MT-I in posterior (A), anterior (B), medial (C), and dorsal (D) views. MT-I has a ginglymoid articulation facet, limiting movement, but increasing strength, contrasting with the more mobile MT-I of *Troodon* sp. (Figure 9). Scale bar  = 2 cm.

In most Cretaceous ecosystems, troodontids lived alongside dromaeosaurids. Divergence of pedal morphology between these sister-taxa potentially affords us insight into variation in their predatory ecology, similar to ecologic separation seen among extant raptor families [19], [20]. In *Troodon* sp. (MOR 553), the distal end of MT-I (Figure 9) is more strongly twisted than that of dromaeosaurids (Figure 10), and it bears a more rounded, ball-like articulation facet (also seen in the more basal troodontid *Sinornithoides* [38]). This suggests greater mobility of D-I in Troodontidae, perhaps allowing D-I to better oppose the other digits. The Late Cretaceous troodontids MOR 553 and *Stenonychosaurus* ([36]; and to a slightly lesser extent *Borogovia* [59]) exhibit shortening of D-IV (by overall shortening of phalanges), which is in contrast with the stratigraphically older (and more basal; [5]) *Sinornithoides* (which has a more elongate D-IV; [38]). This trend is consistent with derived Late Cretaceous troodontids evolving further towards cursorial-adapted feet, while the more basal forms exhibited more grasping ability.

Starting from similar morphology in basal forms (cursorial metatarsus and a grasping foot), dromaeosaurids and troodontids follow opposite morphological trends indicative of increasing ecological separation. Dromaeosaurids appear increasingly adapted for grappling larger prey with strong, but slow feet, and unusually hypertrophied D-II unguals. By comparison, troodontids instead evolved towards a more cursorial habit, being fast and nimble with weaker, but quick feet. The more mobile D-I in troodontids perhaps afforded a more even grip, better adapted for snatching and subduing smaller prey. An important part of our interpretation is that ground-based predation need not necessarily be conducted at high speed. It is commonplace for extant terrestrial predators to employ surprise ambush techniques; goshawks and other forest raptor species commonly hunt on the ground, employing ambush and maneuverability as strategies, rather than outright pursuit [60]. Troodontids exhibit limb proportions consistent with cursorial adaptations, suggesting speed and/or pursuit was important to their predatory strategy. By contrast, derived dromaeosaurids such as *Velociraptor* and *Deinonychus* do not exhibit limb proportions that suggest significant cursorial ability: rather, they were probably more inclined towards utilizing ambush as a main predatory strategy.

In *Deinonychus* and other paravians, grasping adaptations of the digits are not as extremely developed as those seen in extant raptors [15], [20]. Although the flexor tubercles on deinonychosaurian pedal unguals are larger than in other non-avian theropods, the tubercles are still relatively much smaller than those seen in birds of prey. As such, the grip strength of the foot may not have been great enough to constrict small prey as seen in extant raptors. Further, while pedal digits are elongated in deinonychosauria, phalangeal proportions still reflect a degree of cursoriality ([61], i.e. non-ungual phalanges shorten distally, unlike that seen in grasping-adapted birds). This may reflect the fact that the largest deinonychosaurians (and possibly all included taxa) were terrestrial animals, which necessitated a compromise between a locomotor and predatory grasping function.

### Eating, jaws, and teeth

After prey have been captured and immobilized, the task of dismemberment and consumption presents a different challenge to the predator, with important morphological implications. Eating habits vary among birds of prey; owls (small prey specialists) usually swallow prey whole, while falcons and hawks dismember prey before consumption. Prey are typically pinned down between the feet by the claws of both left and right D-II, while D-I, III, and IV contact the ground, steadying the bird for feeding [20]. For larger food items, one or both feet are used in their entirety, which may cause instability, requiring correction by flapping and extending the wings and tail (see Supporting Information Video S2). To feed, the head reaches down between the feet, gripping tissue in the hooked beak, then pulls upwards, plucking away the feathers or tearing off strips of flesh. We envisage deinonychosaurians feeding like accipitrids in holding prey under the feet, with the head reaching down between the feet to feed.

Manning et al. [2], [62] assert the jaws of dromaeosaurids were the main tool in killing their prey, but the mandibles of *Deinonychus*, *Velociraptor*, and *Saurornitholestes* were not particularly robust, lacking a strong bite force [63], [64] and were therefore poorly suited for the primary offense in attack and restraint of large prey. The RPR model better explains this morphology. Here the jaws are employed only for dismemberment; the prey item is typically of a much smaller size, and since it is fully restrained under or within the feet, it subjects the jaws to lower stress when in use. A weak bite force might be considered disadvantageous for a predator; however, accipitrids have a relatively weak bite force (especially compared to other birds of prey [28]), and it does not decrease their predatory effectiveness. Rather, a weaker bite force merely forces accipitrids to adopt a different immobilisation strategy than other raptors [20]. In the absence of any other apparent structure for quick dispatch of prey, it is likely that deinonychosaurians were like accipitrids in simply eating their victims alive.

Head orientation and movement during prey dismemberment may help explain the unusual tooth morphology of Deinonychosauria. Tooth morphology is a conspicuous indicator of diet and feeding strategy. In general, the teeth of theropod dinosaurs are similar in form to extant varanids, being laterally compressed, posteriorly recurved, and possessing denticles on both the anterior and posterior carinae which pinch and tear through flesh, rather than slicing like a knife [65]. The peculiar teeth of Dromaeosauridae (with the possible exception of *Dromaeosaurus*) differ from typical theropods in that the denticles of the posterior carina are particularly elongate, distally hooked towards the tooth apex, and much larger than those of the anterior carina [66]. This character is particularly pronounced in derived Late Cretaceous taxa (e.g., *Velociraptor*, *Saurornitholestes*); indeed anterior denticles may be entirely absent in some *Saurornitholestes* teeth [66], [67]. Denticle reduction on the anterior carina would enhance a piercing function, but the peculiar hooked shape of the posterior denticles would not appear well-suited for tearing through flesh, suggesting behaviour that deviates from more typical theropods. Under the RPR model, hooked posterior denticles may enhance the effectiveness of the jaws' grip on the prey. When the head reaches down between the feet, the jaws become oriented nearly perpendicular to the prey (see Figure 1). With the predator's teeth embedded into its victim, subsequent backward jerking motion of the head (as seen in birds of prey) would pull impaled tissue against the posterior denticles. The denticles' hooked shape potentially enhances grip as tissue is torn away. It is also possible that the peculiar denticles are not an adaptation for hooking flesh, but actually helped in removing feathers or fur from prey items.

The teeth of troodontids are similar to those of dromaeosaurids in that denticles are reduced or absent on the anterior carina, with large hooked denticles on the posterior carina [66], [67]. Troodontid denticles appear proportionally much larger than those of dromaeosaurids, and compared to the crown height this is true. However, troodontids possessed many more teeth in their jaws than would have a similarly sized dromaeosaurid [67]. For a given fixed jaw length, troodontid teeth are comparatively much reduced in size; the crown height of troodontid teeth would have been only about half as much as those of a dromaeosaurid. Therefore it is probably more accurate to say that troodontids do not have large denticles; rather, they have short crowns, with similarly sized denticles as might be expected for a dromaeosaurid of similar body mass. This makes sense if denticles have a size below which they are no longer able to function effectively.

### Low-carried metatarsus helps restrain prey

Evidence from extant acciptrids suggests the metatarsus itself may be used by Deinonychosauria to help restrain prey. Upon contacting with their prey on the ground, the red-tailed hawk (*Buteo jamaicensis*) has been observed bringing the tarsometatarsus into a horizontal position parallel to the substrate [68]. It is not known whether this is typical behaviour for accipitrids as a whole, but it is consistent with video evidence [20]. Footprints show that non-avian theropods carried their metatarsus at a lower angle to the substrate than do extant birds [58]. If *Deinonychus* brought its metatarsus close to the horizontal during prey restraint, this would bring D-I closer to the prey animal, with the metatarsus forming the ‘palm’ of an enclosable fist.

### Mantling and possible use of the forelimb

It remains paradoxical that the manual digits of paravians seem well-suited for flexion and grasping, yet would have borne flight feathers so as to make such an action difficult or clumsy [1], [31], [69]. Further, paravian manual unguals are enlarged and strong expression of flexor tubercles suggests that the claws were capable of exerting considerable force, yet the limited range of motion of the forelimb [31] seems strongly adapted for flapping, rather than the flexibility required for prey manipulation (or indeed, climbing [61]).

Comparison to extant raptors provides a combined functional hypothesis for the forelimbs and unguals that has not been previously considered. During immobilisation, it is common to observe extant raptors encircling their prey with their wings, a posture known as “mantling” [70]. This is observed across all extant raptor families and is thought to either assist in preventing prey escape, or conceals the victim from other predators, lest they attempt piracy. Under the RPR model, if the same strategy was employed by paravians subduing prey, then the large manual unguals may have been used to pull escaping prey back under the feet of the predator in a raking action. This reconstruction lowers the hands to be used near the feet, consistent with the orientation of the palms while in this posture.

### Exceptionally large prey immobilisation strategy

Fossil associations of *Deinonychus* and the ornithischian *Tenontosaurus* (which is of larger body size than *Deinonychus*) have led some workers to hypothesize a predatory relationship between the two, including the possibility of pack-hunting in *Deinonychus* [3]. Coordinated pack-hunting was considered unlikely by Roach and Brinkman [71], although mobbing was thought possible. It is rare to see extant predators taking prey that are significantly larger than themselves; however, Roach and Brinkman note that golden eagles (*Aquila chrysaetos*) have been observed to take down small deer and sheep [72]–[74].

Published accounts of this rare (indeed, disputed) behaviour are anecdotal, but the process by which golden eagles kill large prey is of interest. In most accounts, eagles form a tight fist with their feet, and stoop their prey, striking it at speed [75]. Clearly, this behaviour is not possible for non-volant deinonychosaurians. However, rarer accounts ([76] and references therein) record eagles “prey-riding”; embedding their talons deep into the backs of their much larger prey and holding on as the victim's vigorous retaliations serve only to widen the wounds. Prey-riding can be considered as an extension of the typical accipitrid predatory strategy for dealing with large prey, except that here the victim is too large to be pinned down by the raptor's bodyweight. In order to prevent escape the raptor merely holds on with its hypertrophied talons. Some anecdotal sources suggest that piercing of internal organs by talons hastens the death of the victim (also previously suggested for smaller prey) [68]. Experiment and observation has shown this to be unlikely ([20] and references therein). Instead, the victim is probably immobilized by weakening through exhaustion and/or loss of blood. Prey-riding behaviour was recently filmed for solitary golden eagles attacking reindeer calves [77], and has also been suggested to have been employed by the extinct Haast's eagle, *Harpagornis moorei*: the largest species of raptor known to have existed ([78]; see Supporting Information Text S1). However, prey-riding by eagles is very rarely observed and should not be considered typical. Also, since enlarged D-I and D-II talons are characteristic of all accipitrids [20], most of which have not been observed prey-riding, then this behaviour is probably not a significant selection factor affecting ungual size and morphology.

Prey-riding in eagles is a similar behaviour to the “climbing crampon” hypothesis of Manning et al. [2], [62] whereupon the enlarged D-II claw of deinonychosaurians is suggested to have evolved to maintain purchase on exceptionally large prey. However, as in extant accipitrids, the hypothesis that the hypertrophied D-II claw evolved specifically for this behaviour is unlikely. We do not exclude the possibility that *Deinonychus* and other dromaeosaurids may have successfully attacked prey much larger than themselves, but their anatomy suggests that, as with all known tetrapod predators, they mostly preyed upon animals smaller than themselves.

### Hallucal reversal and evolution of the grasping foot

It has been proposed that the reversal of D-I, the hallux, evolved to grip branches for perching, and as such is an important component of some models for the origin of flight [7], [52], [79]. Although there has been some debate, the hallux of *Archaeopteryx* is now thought to have been medially directed rather than fully reversed ([80]; see Supporting Information Text S1). A fully reversed hallux was reported for the basal bird *Jeholornis* [81], [82], although this is also disputed as all *Jeholornis* specimens are compressed in a fashion similar to *Archaeopteryx* such that apparent hallux reversal may be a preservational artifact. As such, the first appearance of a fully reversed hallux is uncertain, and may not be strictly definable since translocation was probably gradual. Middleton [83] documented the variable position of the hallux in extant birds and concluded by asking which functional changes and selection pressures led to the evolution of a reversed hallux. In contrast to perching-only hypotheses, the RPR model proposes that a grasping foot first evolved for predatory purposes in terrestrial paravians. Selection pressure for increased grasping ability (benefiting predatory success) favored gradual translocation of the hallux to a progressively more reversed position where it could oppose the other digits providing more even grip. This demonstrates a viable selection pathway whereby the necessary grasping ability and hallux reversal required for perching could be exapted from a predatory function in a wholly terrestrial predator, without invoking a hypothetical pre-flight arboreal or scansorial stage for non-avian theropods.

### Grasping for predation or an arboreal habit?

In their description of the small basal dromaeosaurid *Microraptor zhaoensis* (Lower Cretaceous, China), Xu et al. [52] describe a number of pedal characters which they refer to as “consistent with an arboreal habit”: pedal digit I (hallux) is relatively distal in position; pedal unguals show higher curvature than other non-avian theropods; and distal non-ungual phalanges are elongated. Similarly, Feduccia et al. [79] remark that “[hallucal] reversal is an unequivocal arboreal adaptation for grasping branches”. A distally positioned or reversed D-I (hallux) and elongated penultimate phalanges are both features that enhance grasping ability. High ungual curvature enhances the hooking ability of the ungual. These features would all be of considerable use to an arboreal animal, but as shown here and elsewhere ([20] and references therein), they are also proven predatory adaptations. Although these two interpretations are not mutually exclusive, it is a challenge to be able to differentiate them morphologically and elucidate whether one function has been exapted from the other ([61]; a similar problem has been encountered in carnivorous mammals; [9]).

Some features of paravians seem to support the predatory model over the arboreal model, at least as a primary or initial function. The same adaptations for grasping (along with other predatory adaptations) are seen in both small and large bodied deinonychosaurians, including taxa too large to have been arboreal. The subarctometatarsalian condition of the metatarsus, exhibited by basal Deinonychosauria (including *Microraptor*) and derived further in troodontids (fully arctometatarsalian), is an adaptation that affords cursorial benefits: it is difficult to envisage a scenario in which it would be selected for in an arboreal animal. Further, grasping adaptations of the feet are maintained in the otherwise more cursorial troodontids, whose reduced forelimbs (also seen in the basal dromaeosaurid *Tianyuraptor*; [84]) would render them poorly adapted as climbers. It is possible that deinonychosaurians exapted their predatory grasping foot from arboreal ancestors. However, since the ancestors of Paraves were large-bodied terrestrial carnivores, this hypothesis requires basal Paraves to evolve an arboreal habit and adaptations, which are then subsequently lost in Deinonychosauria (potentially also becoming secondarily flightless; [85]). While possible, the multiple behavioural and morphological shifts render this hypothesis less parsimonious than if Paraves were terrestrial carnivores like their ancestors, and the grasping foot evolved initially for predatory purposes being exapted later for perching in Avialae.

### “Stability flapping” and the “flapping first” hypothesis

The origin of the flapping stroke is an important independent step in the origin of flight [86], [87]. Here we describe “stability flapping”, a behavioral component of the RPR model, and propose that it could have been employed by paravians during predatory activity. During the struggle following large-sized prey capture by accipitrids, the hypertrophied D-II talons are locked into the prey, preventing the feet from assisting stabilization of the raptor [20]. To counter this, vigorous stability flapping is typically executed by the raptor in order to first get on top of its prey, then to constantly maintain this position, allowing it to use its full bodyweight to pin its victim to the ground [20] (Supporting Information Videos S1 and S2). If only small corrections are required, the wings are extended for balance with only occasional light flapping, and vertical movement of the tail [20].

**Figure 11:**
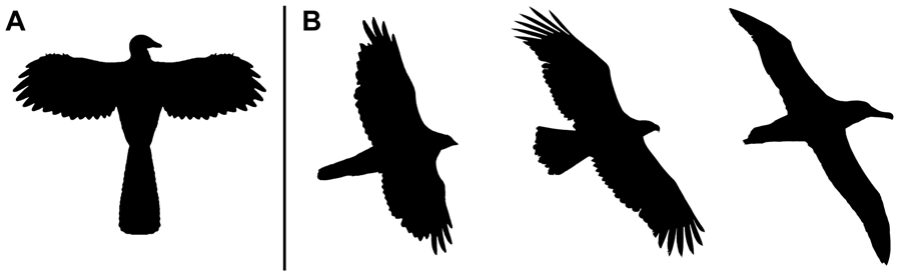
Wing proportions of birds. (**A**) *Archaeopteryx*. (**B**) Variation of wing aspect ratio in extant birds, from left (low) to right (high): goshawk (*Accipiter gentilis*), golden eagle (*Aquila chrysaetos*), northern royal albatross (*Diomedea sanfordi*). The short broad wings of *Archaeopteryx* are similar to the goshawk, where they afford great maneuverability. Image in (**A**) altered from Longrich [101].

Stability flapping supports a “flapping first” model where flapping and associated aerial capability, including generation of lift, can be evolved independently of a flight function. Large feathered wings were present in basal Paraves and Deinonychosauria such as *Archaeopteryx*, *Microraptor*, and *Sinornithosaurus* [41], [52], [88], and the presence of feathered forelimbs in larger species is demonstrated by preserved quill knobs in *Rahonavis* [89], and *Velociraptor* [90]. However, there has been much debate as to the aerial capabilities of these taxa and their importance in the evolution of powered flight. What use is half a wing? Even a relatively small aerofoil and weak flapping capability could be employed for stability flapping, affording a greater chance of predatory success. The low aspect ratio wings seen in *Archaeopteryx* [76] and basal Deinonychosauria [91] are similar in shape to those of extant accipitrines (Figure 11), woodland raptors that capture prey by surprise ambush and frequently utilize stability flapping (Supporting Information Videos S1 and S2; [20]). Short, broad wings confer great maneuverability at a cost of overall speed or soaring ability, and would have been well-suited for stability flapping. Forelimb movement in *Deinonychus* was “comparable to the form of the avian flight stroke” [92], and similar in other basal Paraves and Deinonychosauria ([31], [93]; although Gatesy and Baier [87] questioned the precise similarity between the forelimb movement of *Deinonychus* and that of extant pigeons). Even if Deinonychosauria were not capable of a full avian-like flapping ability, they may have been able to perform a rudimentary flight stroke during stability flapping. Similarly, long feathered tails are conspicuous in accipiters and aid in maneuverability and balance during stability flapping. Basal Paraves and Deinonychosauria possessed long bony tails which are shown to have been well feathered (e.g. [52], [88]), and would have assisted balance during predation [1] and stability flapping.

Stability flapping is less physically demanding than flight, and represents a previously unrecognized intermediate aerial ability. Padian and de Ricqles [94] define four requirements of “flight” and suggest that all are fulfilled in *Archaeopteryx*: (1) an airworthy wing, (2) a flight stroke capable of generating a vortex wake that will propel the animal forward, (3) a metabolic level capable of sustaining flight for substantial intervals, and (4) the neuromuscular coordination that permits effective navigation in a three-dimensional world. Vigorous stability flapping involves most of these requirements, but each can be functional in a less developed state than is necessary for flight. Hence, stepwise acquisition and development of Padian and de Ricqles' flight requirements might have been facilitated by gradual evolution of less energetic behaviours leading to stability flapping. The presence of feathered forelimbs is well documented even in taxa basal to Paraves (e.g. the oviraptorosaurians *Caudipteryx* and *Protarchaeopteryx*, and therizinosaurosauroid *Beipiaosaurus*; [5], [51], [95]). Few would suggest that these are airworthy wings, but they may have provided some aid to balance (even outside of a predatory role). It is conceivable that vigorous stability flapping evolved from the simple outstretching of forelimbs for balance, developing through an intermediate stage consisting of short flaps and tail movement. Both of these behaviours are often employed by extant birds of prey for small positional corrections [20]. The step from stability flapping to powered flight requires significant generation of forward thrust. Although directional thrust is employed for positional changes during stability flapping, it is not yet clear how this might be adapted into a method of propulsion.

Stability flapping (and other flapping behaviours [96], [97]) would have been most effective at small body sizes and might have been a factor driving selection for miniaturization in Coelurosauria [94], [98]. The “flapping first” model provides a viable selection pathway whereby decrease in body size through Coelurosauria is associated with increase in flapping adaptations of the forelimbs, culminating in small body-size at the base of Paraves [94], [98]. Thus, *Deinonychus*, *Velociraptor*, and other relatively large-bodied deinonychosaurians were probably derived from small-bodied ancestors. Even if stability flapping does not represent a condition ancestral to true flapping flight, it may help explain the prevalence of apparent flapping and aerial abilities in otherwise terrestrial taxa. The presence of secondary flight feathers on the forelimbs of *Velociraptor* [88] might be unexpected since the large body size of this taxon appears to preclude a flighted or gliding function (although *Velociraptor* was of similar mass to the largest extant flying birds, e.g. bustards at ∼19 kg [99]). However, stability flapping (especially in its less vigorous forms) may still be a viable use for a wing, even in a taxon as large as an adult *Velociraptor*.

### Conclusions

The Raptor Prey Restraint (RPR) model presents multiple new concepts that give functional explanations for the morphological peculiarities of *Deinonychus* and other paravians. These findings open many novel lines of research into the predatory ability of extinct theropods, and emphasize the importance of exaptation in the evolution of novel structures and behaviours. The hypertrophied D-II talon of Accipitridae represents the closest analogue yet presented for use of the similarly hypertrophied D-II talon of Deinonychosauria. Gradual divergence of foot proportions within Deinonychosauria is potentially indicative of ecological separation between Dromaeosauridae and Troodontidae as large and small prey specialists (respectively). Future research on the diet of extinct theropods should include analysis of foot functional morphology, which has the potential to test hypotheses recently presented by Zanno and Makovicky [44]. The grasping foot of paravians demonstrates a shift in emphasis for prey restraint from the manus to the pes, as the forelimbs became increasingly feathered and adapted for flapping functions through Coelurosauria.

In our description of stability flapping and its importance to predatory success, we hope to have opened a new direction of study in the evolution of flight in birds. The RPR model demonstrates that there need not be a scenario where flight is gained (and lost) numerous times [85]. Rather, we present the more parsimonious “flapping first” hypothesis: that basal paravians exhibited a range of flapping behaviours unrelated to flight [96], [97], but that it was only in Avialae where true flapping flight evolved as a method of aerial locomotion.

A more precise definition of stability flapping is in preparation such that future studies can better focus on potential osteological or biomechanical correlates. Further investigation is also required into other flapping behaviours that do not involve flight, including stability flapping executed outside of a predatory role. Hence, much work remains in characterizing stability flapping, but as with other recently proposed models [96], [97], recognition of this novel behaviour enriches our understanding [94] of the physical capabilities of the ancestors of modern volant birds.

## Supporting Information

Text S1 Text S1 includes additional discussion of previous work on paravian claw morphology and function, comparison of Deinonychosauria with extant seriema birds, reconsideration of the origin of the avian pes Tendon Locking Mechanism, and the possibility of stability flapping in Haast's eagle: an extinct giant accipitrid.
(DOC)

Video S1 **Stability flapping in a wild Eurasian Sparrowhawk (** ***Accipiter nisus*** **).** The vigorous flapping of this sparrowhawk is not an attempt to fly away with its prey. Rather, this is “stability flapping”: employed only to get on top of the prey and maintain this position so that the raptor can use its bodyweight to pin down its victim. With the feet employed in preventing escape, the forelimbs must now be used to maintain an advantageous position: the opposite to what is seen in basal theropods where the forelimbs presumably had a greater role in subduing prey, with the feet used for positioning. Filmed 12^th^ March 1998, Nacton, Suffolk, UK.
(AVI)

Video S2 **Prey positioning in a wild Eurasian Sparrowhawk (** ***Accipiter nisus*** **).** Here the sparrowhawk has the prey pinned between its D-II talons, with the other toes used for stable footing. Even though its victim is still alive, the raptor can continue to feed as the prey is well restrained by the predator's bodyweight and claws. Light stability flapping is intermittently employed to maintain position. Filmed 12^th^ March 1998, Nacton, Suffolk, UK.
(AVI)

Table S1 This table comprises raw measurement data and calculated ratios for dinosaurs measured for this study, and comparative data from Fowler et al. [20].
(XLS)
