# Supplementary material for: The Predatory Ecology of Deinonychus and the Origin of Flapping in Birds
Source: PLoS One. 2011 Dec 14;6(12):e28964. doi: 10.1371/journal.pone.0028964 (PMC3237572; doi:10.1371/journal.pone.0028964)
Supplement: Text S1 — includes additional discussion of previous work on paravian claw morphology and function, comparison of Deinonychosauria with extant seriema birds, reconsideration of the origin of the avian pes Tendon Locking Mechanism, and the possibility of stability flapping in Haast's eagle: an extinct giant accipitrid. (DOC) [file pone.0028964.s001.doc]

# The predatory ecology of *Deinonychus* and the origin of flapping in birds

Fowler, D. W.^1^; Freedman, E. A.^1^; Scannella, J. B.^1^; Kambic, R. E.^2^

^1^ Museum of the Rockies & Department of Earth Sciences, Montana State University, Bozeman, Montana

^2^ Brown University, Providence, Rhode Island

# Supporting Information Text S1

## Contents

1. Previous work

1.1 Manning et al. (2006; 2009)

1.2 Feduccia et al (2007)

1.3 Zanno and Makovicky (2011)

2. Supplemental Discussion

2.1 *Archaeopteryx* outside of Aves

2.2 Scansoriopterygidae

2.3 Wing Assisted Incline Running.

3. Other comparisons and wider implications

3.1 Comparison to the seriema birds

3.2 Origins of the tendon locking mechanism in birds

3.3 Stability flapping in Haast’s eagles

4. References

## 1. PREVIOUS WORK

**1.1 Manning et al (2006; 2009)**

In a novel experiment, and the only real attempt to elucidate a function for the hypertrophied D-II claw of deinonychosaurians, Manning et al. (2006) constructed a model of the *Deinonychus* D-II claw and simulated attacks on pig and crocodile skin. They concluded that the hypertrophied D-II claw would not be effective as a slashing device and suggested instead that it was used by dromaeosaurids as a climbing crampon for gripping the hides of prey several times larger than themselves.

In 2009, Manning et al. expanded upon their 2006 paper by conducting Finite Element Analysis (FEA) of a single *Velociraptor* manus ungual (LL.12392; Manchester Museum, University of Manchester, UK) utilizing input data from the pes claw of an eagle owl (*Bubo bubo*). From their analysis of the manual ungual, they then transferred their functional interpretation to the hypertrophied D-II ungual of the pes. From this, they proposed that the claws of dromaeosaurids were “well-adapted for climbing as they would have been resistant to forces acting in a single (longitudinal) plane, in this case due to gravity.”

Given that the conclusions of our study take a mostly opposing view to those of Manning et al. (2006, 2009), we feel it is appropriate to discuss these papers in greater detail, highlighting what we believe to be methodological error and misinterpretation of data.

In Manning et al. (2006) the shape of their mechanical model D-II *Deinonychus* claw may have been reconstructed incorrectly. The ventral surfaces of *Deinonychus* unguals D-I, III, and IV are flat to convex, as are all unguals in extant acciptrids (Fowler et al., 2009), and when combined with a keratinous sheath would likely have produced a similar arched cross-section (Manning et al., 2006). However, *contra* Manning et al. (2006), in all deinonychosaurian specimens studied in our analysis the D-II ungual possesses a weakly keeled ventral surface, suggesting that the keratinous sheath probably was not so arched in cross section. Manning et al. (2006) noted that some owls have a more tubular ungual cross section, which we also note for the osprey (which has laterally compressed claws on all its digits; Fowler et al., 2009). Since the *Deinonychus* model D-II talon of Manning et al. (2006) was reconstructed as arched, this probably limited its slashing capability.

Manning et al. (2006) used the D-II ungual of the *Deinonychus* holotype YPM 5205 (Peabody Museum of Natural History, Yale University, New Haven, Connecticut; Ostrom 1969) as the basis of their model, but this particular specimen is unusually highly curved (our measurement: Oo = 152.7^o^; the highest for any measured theropod), even compared to all other measured deinonychosaurian D-II unguals (n = 15; Oo = 94.5^o^ to 130.9^o^; mean = 111.6^o^; median = 108.9^o^). Indeed, Ostrom (1976) figured a second specimen of near identical size (MCZ 4371) and noted its much lower curvature (Oo = 107.5^o^; our measurement), which falls within our expected range for Deinonychosauria. Given the aberrant curvature of YPM 5205, it would have been appropriate to have chosen a more typical specimen for the mechanical testing. It is not clear as to the extent to which the much higher curvature of YPM 5205 affected the results of Manning et al.’s (2006) experiment, but it does have significant bearing on the conclusions of the follow-up study (Manning et al., 2009).

Much of the evidence supporting a climbing function for the D-II ungual depends upon erroneous interpretation of claw curvature. Manning et al. (2009) state that “claw geometry of mammals and birds correlates well with arboreal and terrestrial habits”, then cite the controversial paper of Feduccia (1993). Feduccia’s (1993) work has been revisited numerous times, most recently by Glen and Bennett (2007). They critiqued Feduccia’s rigid ecological categorisation, suggesting instead a series of more intermediate ecological groups, and also noted that Feduccia’s choice of measured curvature (inner curvature) was problematic in that the inner curve is highly variable and that Feduccia’s method of measurement incorporates part of the plantar surface of the toe pad (including the flexor tubercle, which tends to be enlarged in predatory taxa, hence their curvature may be overestimated). As a consequence, Feduccia’s method tends to give a higher measure of inner claw curvature. Correcting for these errors, Glen and Bennett then similarly compared extinct to extant taxa, based on D-III outer curvature (a more reliable measure than the inner curvature used by Feduccia), and found that basal birds (with the possible exception of *Sapeornis*) and non-avian theropods had claw curvature consistent with ground foraging, not an arboreal habit. Manning et al. (2009) do not cite Glen and Bennett’s paper (perhaps due to timing of submission dates), nor any other previous work (other than Feduccia, 1993) on the curvature, effectiveness and use of bird claws, (e.g. Peters and Gorgner, 1992; Pike and Maitland, 2004; Csermely and Rossi, 2006; Einoder and Richardson, 2007). The work of Pike and Maitland (2004) is especially relevant since it shows that in birds, claw curvature scales with body mass. Since *Deinonychus* and *Velociraptor* are many times larger than the birds with which they are being compared, higher claw curvature might be wholly expected if they follow the same scaling patterns. We suspect that had these publications been consulted, Manning et al. (2009) would have interpreted their data differently, and come to a conclusion closer to our own.

Nevertheless, Manning et al. (2009) use Feduccia’s (1993) ecologic categories to infer an arboreal habit for all dromaeosaurids, basing their assessment on a single measured specimen: the D-II pes ungual of the *Deinonychus* holotype YPM 5205, which as mentioned above, is aberrant, being significantly more curved than all other deinonychosaurian D-II unguals measured by us. Manning et al. took their curvature value of 160^o^ for YPM 5205 directly from the original description by Ostrom (1969) rather than re-measuring it using Feduccia’s method (which is the method they used to measure the *Velociraptor* and *Bubo* claws), thus the data are not properly comparable. We repeated measurement of the claws from Manning et al. (2009) using Feduccia’s method, resulting in an inner curvature of 124^o^ for the manus ungual of *Velociraptor* (LL.12392) and 174^o^ for the pes D-II ungual of *Deinonychus* (YPM 5205). Accounting for some additional curvature (up to 15%, Manning et al., 2006) provided by the keratin sheath, these measurements would seem to fit into Feduccia’s (1993) range for trunk-climbers: 129.5-161.6^o^ (although it should be noted that *Velociraptor* is borderline, lying close to the perching category: 101.8^o^ – 125.3^o^). However, Feduccia’s categories were concerned only with D-III claws. Manning et al. (2006, 2009) do not mention this, yet it is vitally important to the interpretation of their data. Indeed, if we took the D-II data for birds of prey (Fowler et al., 2009), and input it into Feduccia’s categories (even without re-measuring using Feduccia’s method), birds of prey would also be considered as trunk climbers. This demonstrates that applying categories of D-III curvature to D-II (as do Manning et al., 2009) is unreliable and likely to yield incorrect results. Thus the conclusion of Manning et al. (2006, 2009) that D-II curvature is indicative of a climbing habit for dromaeosaurids is, in fact, not supportable by their data.

To test the actual placement of *Deinonychus* in Feduccia’s categories, we re-measured the curvature of the YPM 5205 *Deinonychus* pes D-III ungual using Feduccia’s method. We found the curvature of 92.5^o^ falls between Feduccia’s ground and perching categories (similar to the findings of Glen and Bennett, 2007). Even allowing for up to 15% extra curvature from a keratin sheath, this figure does not fall into the climbing category. This highlights a deeper methodological error, that at no point do Feduccia (1993) nor Manning et al. (2009) make any comparisons to birds of prey (indeed, Feduccia specifically excludes them). This is especially important for elucidating claw use in dromaeosaurids as they are considered carnivorous, and it is surprising that this was overlooked by Manning et al. (2009), especially since they use an owl claw to help model their input data for the FEA analysis. Clearly, a predatory function for the claw severely affects its curvature (although see later): generally speaking, active predators have more curved claws than non-active predators (Fowler et al., 2009).

However, despite our many disagreements with the source materials, methods, and interpretations of Manning et al. (2006, 2009), we agree with their conclusion that the hypertrophied claw of pes D-II would be ineffective as a slashing weapon. Similarly we agree that it was most likely used as a hooked device. However, from the evidence presented here, we reject their suggestion that climbing would be a primary function, as it is unsupported by their own data, and that represented by our analyses (Fowler et al., 2009; this article).

**1.2 Feduccia et al. (2007)**

The arguments of Feduccia et al. concerning the evolution of birds and flight have been commented upon at length elsewhere (e.g. Padian and Horner, 2002), and further discussion contributes little to knowledge regarding this important transition. However, Feduccia et al. (2007) draw attention to some aspects of claw curvature, morphology and hallucal orientation in their discussion of the description of the Thermopolis specimen of *Archaeopteryx* (Mayr et al., 2005; 2007), and we feel it is appropriate to address some of these comments here.

In response to the observation by Mayr et al. (2005; 2007), that the hallux of the Thermopolis *Archaeopteryx* is not reversed but instead is directed medially, Feduccia et al. state that “this position is dubious for biomechanical reasons and would certainly inhibit terrestrial locomotion” and that “we are unaware of such a positioning in the pes of other animals”, yet this is precisely the orientation that the hallux takes in *Deinonychus*, and *Velociraptor*, both given as examples of terrestrial animals elsewhere in their own manuscript. Indeed, in their figure 2, they show a photograph of a *Velociraptor* pes in which the condyles of the distal end of MT-I face medially, as also seen in 3D articulated *Velociraptor* specimens figured by Norell and Makovicky (1997; 1999) and potentially the recently described *Balaur* (Csiki et al., 2010). Furthermore, a medially directed D-I appears to be a character shared by even more basal theropods. For example, in three-dimensionally preserved articulated specimens it can be seen that the basal ornithomimosaur *Garudimimus* has a medially directed D-I (Kobayashi and Barsbold, 2005) as does the large alvarezsaurid *Kol ghuva* (Turner et al., 2009). Manipulation of fossil specimens shows that a medially directed D-I is lesser developed, yet still present, at least as basally as *Allosaurus* and *Tyrannosaurus* (MOR 693 and MOR 555, respectively; Museum of the Rockies, Bozeman, Montana). Furthermore, footprint evidence shows a medially to anteromedially directed D-I in Upper Triassic theropods (Gatesy et al., 1999) thus demonstrating that D-I becomes progressively more medially directed through theropod evolution. Admittedly, the D-I of all non-avian theropod taxa is not “reversed” as seen in many (but not all) extant birds, but considering only “reversed” or “unreversed” as two discrete and unconnected stages is a typological argument and represents poorly the full spectrum of angles at which D-I is carried (e.g. Middleton, 2001). Indeed, Mayr et al. (2005; 2007) paid special attention to the hallucal orientation of *Archaeopteryx*, precisely because of this apparent morphological continuum, and because their specimen provided new details which would help to resolve the long debate surrounding hallucal orientation in *Archaeopteryx*. If the phylogenetic position of *Archaeopteryx* within Coelurosauria is accepted, then a medially directed hallux is expected, rather than being an unusual condition. Feduccia et al.’s criticism of Mayr et al is thereby shown to be invalid.

Feduccia et al. (2007) assert that claw curvature is “a definitive marker of arboreal habit”, citing Feduccia (1993), and suggest that this was ignored by Mayr et al (2005; 2007). As shown in the previous section, Feduccia’s (1993) method of analysis was criticized by Glen and Bennett (2007), and also excluded predatory birds from the dataset, making any comparison to predatory non-avian dinosaurs (including *Archaeopteryx*) problematic. Aside from Glen and Bennett’s (2007) assertions, problems with linking claw curvature and behaviour are illustrated in our sibling study (Fowler et al., 2009), where we describe variation in claw morphology and predatory strategy in extant birds of prey. Usually, predatory birds possess claws with very high curvature (enough for them to fit into the trunk climber category of Feduccia, 1993), but owl talons are typically only weakly curved. This aberration only makes sense when considered alongside their short toes and tarsometatarsus, increasing leverage (and hence strength) in the feet (Ward et al., 2002). Owl claws are large with low curvature so that they have additional length to compensate for the short toes, maintaining reach of each digit (for a more detailed explanation see Fowler et al., 2009). From this example and the overlap of trunk-climbing non-predatory birds with non-trunk-climbing predatory birds, claw curvature alone is not completely reliable in elucidating ecological categories.

Feduccia et al. (2007) also remark that “[hallucal] reversal is an unequivocal arboreal adaptation for grasping branches”. We disagree. Hallucal (D-I) reversal is an adaptation for grasping, but one of the principal findings of our analysis is that a grasping foot might have evolved for purposes other than perching on branches. This is further discussed in the main manuscript.

**1.3 Zanno and Makovicky (2011)**

Zanno and Makovicky (2011) suggested that herbivory was widespread among coelurosaurian non-avian theropods (including at the base of Paraves), and that carnivory in Deinonychosauria was potentially secondarily derived. Zanno and Makovicky define “herbivore” as “species obtaining a high percentage of their diet from herbaceous fodder” and “do not draw a hard distinction” between herbivory and omnivory (Supporting Information). Thus their “herbivory” actually describes varying degrees of omnivory (as seen in most extant birds) rather than herbivory in the traditional sense (which is exceptionally rare in extant archosaurs).

We agree that the available evidence supports either herbivory or omnivory in Ornithomimosauria, Alvarezsauroidea, Therizinosauria, and Oviraptosauria, and that herbivorous adaptations increase from basal to derived taxa within these clades. However, the evidence for herbivory as a basal trait for Coelurosauria or Paraves is currently not well-supported, and we do not think that there is any good reason to consider carnivory as secondarily derived in Deinonychosauria. The Jurassic basal coelurosaurs Tyrannosauroidea, Compsognathidae, and *Ornitholestes* are not considered as herbivorous (Zanno and Makovicky, 2011). Senter (2007) and Zanno and Makovicky (2011) show *Ornitholestes* as more derived than Ornithomimidae, which suggests either secondarily derived herbivory in Ornithomimidae, or secondarily derived carnivory in *Ornitholestes*.

Zanno and Makovicky (2011) suggest that early evolution of a beak in Coelurosauria correlates with a herbivorous diet. Although correlation of a beak with herbivory is well-supported, evidence strongly suggests that a beak was independently acquired in “herbivorous” coelurosaurian clades. Basal members of the Ornithomimidae (*Pelecanimimus*; Perez-Moreno et al., 1994) and Oviraptoridae (*Incisivosaurus*; Xu et al., 2002; Balanoff et al., 2009), exhibit teeth in the premaxilla, and thus probably lacked beaks. The basalmost therizinosaurid, *Falcarius*, exhibits teeth in the maxilla and dentary, but the condition of the premaxilla is unknown (Zanno, 2010). Similarly, potentially tooth-bearing portions of Alvarezsaurid premaxillae are not preserved, but teeth are present in the maxilla and dentary (Chiappe et al., 2002).

We consider it likely that coelurosaurs basally were carnivorous, and that various degrees of omnivory led to increased reliance on vegetative matter in the Ornithomimosauria (potentially independently, if the phylogeny of Senter, 2007, and Zanno and Makovicky, 2011, are correct), Alvarezsauroidea, Therizinosauria, and Oviraptosauria.

In the main manuscript, we indicate that morphological divergence within Deinonychosauria is indicative of large-prey specialist behaviour in dromaeosaurids, and smaller average prey size in troodontids. In Troodontidae, the retention of predatory features of the pes (enlarged D-II ungual), and the further evolution of grasping characters (increased mobility of D-I) suggest at least occasional carnivory, although this may have been only part of a more omnivorous diet, as has been suggested previously for *Troodon* (Holtz et al., 1998; Zanno and Makovicky, 2011).

Finally, we found that pedal characters indirectly suggest a non-carnivorous diet for ornithomimids and alvarezsaurids (see main manuscript). Detailed investigation of pedal proportions in other clades may provide indirect indication of diet, potentially testing the findings of Zanno and Makovicky (2011).

## 2. SUPPLEMENTAL DISCUSSION

**2.1 *Archaeopteryx* outside of Aves**

Two recent phylogenetic analyses placed *Archaeopteryx* outside of Aves as a basal dromaeosaurid, or basal paravian (Xu et al., 2011; Naish et al., 2011; respectively). Neither repositioning represents a problem for the RPR model and the implications for the origin of flight as a basal paravian (Naish et al., 2011) or dromaeosaurid (Xu et al., 2011) position for *Archaeopteryx* is consistent with the similarity of its pes functional morphology with basal deinonychosaurians (subarctometatarsalian metatarsus, elongate D-IV, enlarged D-II ungual, etc).

**2.2 Scansoriopterygidae.**

The Scansoriopterygidae are a clade of unusual theropods, known from only three specimens (*Scansoriopteryx heilmanni*, CAGS02-IG-gausa-1/DM607, China Academy of Geological Sciences, Czerkas and Yuan, 2002; *Epidendrosaurus ningchengensis*, IVPP V12653, Institute of Vertebrate Paleontology and Paleoanthroplogy, Beijing, China, Zhang et al., 2002; and *Epidexipteryx hui*, IVPP V15471, Zhang et al., 2008), all from the Middle to Late Jurassic Dahougou beds of Nei Mongol, China (Wang et al., 2005), and all representing immature individuals. Most recent phylogenetic analyses have placed the Scansoriopterygidae as the sister group to Avialae (Senter, 2007; Zhang et al., 2008; Choiniere et al., 2010), as such some short discussion is appropriate.

The Scansoriopterygidae exhibit aberrant morphology compared to other paravians; an especially elongate manual D-III, relatively long forelimbs, and proportions of the pedal phalanges have been interpreted as adaptations for a scansorial or arboreal habit (Zhang et al., 2002; Czerkas and Yuan, 2002). However, the manual unguals are less curved than in other maniraptorans (Zhang et al., 2008), which might not be expected if the forelimbs were primarily adapted for climbing, or a predatory function. Without any further detailed analysis of their functional morphology, the ecological habit of Scansoriopterygidae is far from certain.

The flapping first hypothesis is not necessarily exclusive of climbing adaptations in basal birds and/or Scansoriopterygidae. We propose that flapping behaviours were utilized for a variety of functions in coelurosaurs, and that only in Avialae was this exapted into a method of aerial locomotion (flight; see main manuscript). If some clades of paravians explored scansorial or arboreal niches through climbing (although evidence is currently lacking), then these taxa may have similarly utilized flapping behaviours derived from those used by their ancestors. However, despite their phylogenetic position, the aberrant morphology of Scansoriopterygidae (compared to other Paravians) may place them as an experimental side-step in the evolution of Paraves.

It remains to be seen how much of the aberrant morphology and phylogenetic position of Scansoriopterygidae is due to the ontogenetic status of the described specimens (e.g. the forelimbs may lack flight feathers in *Epidexipteryx*; Zhang et al., 2008; juvenile dinosaurs often occupy a different phylogenetic position from adults of the same species; Rozhdestvensky, 1965; Fowler et al., 2011; Tsuihiji et al., 2011). Further, the phylogenetic position of such a newly described, unusually derived, morphology is often unstable (as was the case when Alvarezsauridae initially grouped within Aves; Chiappe et al., 1996; Sereno, 2001), and at least one analysis has shown Scansoriopterygidae as the sistergroup to Oviraptorosauria (Xu et al., 2010). Nevertheless, the new directions for research that we outline here may help shed new light on the habits and evolutionary trends of Scansoriopterygidae.

**2.3 Wing Assisted Incline Running.**

Stability flapping is entirely complimentary to other extant bird behaviours that have been proposed as a possible analogue for the origin of flapping. This includes Wing Assisted Incline Running (WAIR), where ground-dwelling chukar partridges were observed running up an inclined surface flapping vigorously in order for their feet to gain better purchase (Dial, 2003; Dial et al, 2008). We foresee no problems with models of flapping evolution that might incorporate both WAIR and stability flapping, based as they are on observed behaviours in extant taxa, rather than hypothetical models.

### 3. Other comparisons and wider implications

**3.1 Comparison to the seriema birds**

Some comparison has been made between dromaeosaurids and the extant seriema birds (Cariamidae: *Cariama* *cristata*: crested seriema; *Chunga burmeisteri*: black-legged or Burmeister’s seriema), as both possess an enlarged ungual on pes D-II. Seriemas are medium to large (*Cariama* stands about 90cm tall) long-legged ground birds native to South America, living in woodland or grassland, and feeding on fruits, insects, and small vertebrate prey (Miranda-Ribeiro. 1937; Burmeister, 1937; Sick, 1993). While the terrestrial habits and hooked D-II ungual appear favorable for initial comparison, further investigation reveals that dromaeosaurids and seriema birds are dissimilar in most aspects of their pes anatomy.

We studied two specimens (AMNH 1392, female: one foot; AMNH 12374, male: both feet; American Museum of Natural History, New York), four photographs, and four published figures (Miranda-Ribeiro, 1937; Burmeister 1937) of *Cariama cristata* feet. AMNH 1392 was precisely measured and included in the dataset for Fowler et al. (2009).

Firstly, the D-II talon of AMNH 1392, while certainly exhibiting unusually high curvature, is only slightly larger (4%) than the D-III claw (an observation confirmed from other specimens and photos). While this is within a typical range for falconids, and higher than expected for passerines, it is hardly similar to the hypertrophied condition seen in Accipitridae (n=17, mean=36.5% larger, s.d.=16.8%; Fowler et al., 2009) and Dromaeosauridae (n=7, mean=57.8% larger, s.d.=24.6%). Indeed, the seabirds puffins (*Fratercula*) and skuas (Stercorariidae) also possess an unusually hooked, yet not significantly enlarged claw on D-II (Peters & Gorgner, 1992), yet there have been no comparisons of these taxa with dromaeosaurids.

Furthermore, our observations found that the proportions of the *Cariama* hindlimb and pes are quite unlike dromaeosauridae, and typical of a cursorial bird, with an extremely elongated metatarsus, outer digits (especially D-IV) much shorter than D-III, shortened distal phalanges, and lengthened proximal phalanges. These findings are in agreement with other reports (Burmeister, 1937; Sick, 1993; Hallager, 2004) where the authors conclude that the short toes of seriemas are of little use in grasping prey. Instead, seriemas either hold prey in their beaks and beat them against the ground, or use their beaks to dismember their prey while it is pinned under the feet (Boyle, 1917; Burmeister 1937; Redford & Peters, 1986; Sick, 1993; Hallager, 2004), employing the D-II claw in a similar fashion to the pinning of small prey by accipitrids (Fowler et al., 2009). In this sense, use of the D-II talon by Seriema birds is similar to part of what we propose for Deinonychosauria, further illustrating that more hooked claws may be associated with active predation (although there are exceptions, see main manuscript section on owls).

Seriema D-II claws have also been observed being used to help gain purchase on tree trunks when the bird is finding a roosting spot, or returning to its nest (Sick, 1993). From this, it might be suggested that the claw serves in some small amount as an adaptation to trunk climbing. However, while some birds (such as woodpeckers) are clearly specialized for the task, all birds use their claws to aid in climbing, so the fact that *Cariama* does so is uninformative.

Interestingly, stability flapping has been previously observed for *Cariama*. Redford and Peters (1986) record “an aggressive encounter between two seriemas that repeatedly jumped at one another, feet first, flapping their wings for balance”. This is not unexpected, since many birds engage in stability flapping to aid balance and positioning when their feet are engaged in some other activity.

Finally, it is worthy of note that despite the enlarged D-II claw typically being carried raised above the ground, a shallow D-II claw impression is preserved in the only published footprints of a seriema (*Chunga burmeisteri*; Gaston, 2003).

**3.2 Origins of the tendon locking mechanism in birds.**

The tendon locking mechanism (TLM) of birds is a ratchet-like adaptation of the feet that allows them to lock in a flexed position without the need to maintain flexion of the flexor tendons (Quinn and Baumel, 1990). A similar mechanism has also independently evolved in Chiroptera (bats), Dermoptera (flying lemurs), and at least two species of climbing rodent (Simmons and Quinn, 1994; Haffner, 1999).

The TLM is used by extant birds in a variety of ways. Most perching birds use the TLM to lock the feet closed to maintain grip on a perch without the need to exert constant muscular effort. This is especially useful in staying perched while the bird is sleeping. Many water birds (such as Anseriformes) employ the TLM to maintain a “cupped” shape of the foot, to aid in swimming (Quinn and Baumel, 1990). In predatory birds, The TLM is employed to maintain grip on prey (Ward et al, 2002; Einoder and Richardson, 2006).

Ward et al (2002) suggest that the modified TLM of predatory birds was exapted from the system seen in normal perching birds. However, if our hypothesis about predatory grasping adaptations in basal paravians is correct, then it presents the intriguing possibility that the TLM may have originally evolved for predatory purposes in paravians, later being exapted (and possibly further refined) for perching in more derived birds. Certainly, such an adaptation would have been advantageous to predatory basal paravians, but due to a lack of an osteological correlate it is not possible to indicate directly the presence of a TLM based on bones alone. However, there is some indirect evidence to support this hypothesis. Manning et al (2009) note that a robust longitudinal ridge is present on the metatarsus of the dromaeosaurids *Velociraptor* and *Deinonychus*, and the troodontid *Sinornithoides*. They suggest that this might correlate to the medial plantar crest of the tarsometatarsus of birds, which is important for the attachment and partitioning of the powerful flexors. At the least, this is in support of the anatomical basis for a strong grip being present in deinonychosauria, and raises the possibility of a TLM also being present.

From a phylogenetic perspective (Quinn and Baumel, 1990), a TLM is present in all extant avian clades with the exception of palaeognaths and Sphenisciformes (penguins). The absence of a TLM in palaeognaths might be due to their basal position within Aves, thereby inferring that a TLM first appeared later, in more derived taxa. However, the absence of a TLM in the relatively derived Sphenisciformes (whose closest relatives, including more basal clades, all possess a TLM), and selected taxa among other clades of Aves, reinforces the hypothesis that secondary loss of a TLM is commonplace, especially among those taxonomic groups for which it would provide little benefit (Quinn and Baumel, 1990). Taking this perspective, palaeognaths might have independently lost their TLM since their pes anatomy has evolved away from a grasping function towards a dominantly cursorial habit. Therefore, at least for the time being, the possibility that a TLM of sorts was present in basal paravians cannot be falsified, and we do not see any reason to suppose preferentially that the TLM evolved first for perching, rather than predation or even swimming.

**3.3 Stability flapping in Haast’s eagles**

Bunce et al. (2005) propose that Haast’s eagles rode the backs of their moa prey, leaving distinctive puncture marks in the pelvises of their victims. Haast’s eagles exhibit other physical traits supportive of this predatory behaviour: they are reconstructed as possessing short, broad (low aspect ratio) wings (Brathwaite, 1992). While this was previously mischaracterised as suggestive of a trend towards flightlessness (see discussion in Brathwaite, 1992 and references therein), short broad wings confer additional maneuverability at the cost of maximum speed, and characterize extant accipitrine accipitrids which employ stability flapping while subduing prey (Fowler et al., 2009).

**4. References**:

Balanoff, A. M., X. Xu, Y. Kobayashi, Y. Matsufune, and M. A. Norell. 2009. Cranial osteology of the theropod dinosaur *Incisivosaurus gauthieri* (Theropoda: Oviraptorosauria). American Museum Novitates 3651:1-35

Boyle, H. S. 1917. Field notes on the seriema (*Chunga burmeisteri*). Auk 34:294-296.

Brathwaite, D. H. 1992. Notes on the weight, flying ability, habitat, and prey of Haast's eagle. Notornis 39:239-247.

Bunce, M., M. Szulkin, H. R. L. Lerner, I. Barnes, B. Shapiro, A. Cooper, and R. N. Holdaway. 2005. Ancient DNA Provides New Insights into the Evolutionary History of New Zealand's Extinct Giant Eagle. PLoS Biology 3(1): e9. doi:10.1371/journal.pbio.0030009

Burmeister, H. 1937. Contribuicao Para a Historia Natural da Seriema (Contribution to the Natural History of the Seriema). Revista do Museu Paulista 23:91-152.

Chiappe, L. M., M. A. Norell, and J. M. Clark. 1996. Phylogenetic position of *Mononykus* (Aves: Alvarezsauridae) from the Late Cretaceous of the Gobi Desert. Memoirs of the Queensland Museum 39:557-582.

Chiappe, L. M., M. A. Norell, and J. M. Clark. 2002. The Cretaceous, short-armed Alvarezsauridae: *Mononykus* and its kin; pp. 87-120 in L. M. Chiappe and L. M. Witmer (eds), Mesozoic Birds: Above the Heads of Dinosaurs. University of California Press, Berkeley.

Choiniere, J. N., X. Xu, J. M. Clark, C. A. Forster, Y. Guo, and F. Han. 2010. A basal alvarezsauroid theropod from the Early Late Jurassic of Xinjiang, China. Science 327: 571–574.

Csermely, D, and O. Rossi. 2006. Bird claws and bird of prey talons: Where is the difference? Italian Journal of Zoology 73:43-53.

Czerkas, S.A., and C. Yuan. 2002. An arboreal maniraptoran from northeast China; pp. 63-95 in S. J. Czerkas (ed), Feathered Dinosaurs and the Origin of Flight. The Dinosaur Museum Journal 1. Blanding, Utah.

Dial, K. P. 2003. Wing-assisted incline running and the evolution of flight. Science 299:402-404.

Dial, K. P., B.E. Jackson, and P. Segre. 2008. A fundamental avian wing stroke provides a new perspective on the evolution of flight, Nature 451:985-988.

Einoder, L., and A. Richardson. 2006. An ecomorphological study of the raptorial digital tendon locking mechanism, Ibis 148:515-525.

Einoder, L., and A. Richardson. 2007. Aspects of the hindlimb morphology of some Australasian birds of prey: a comparative and quantitative study. Auk 124:773-788.

Feduccia, A. 1993. Evidence from claw geometry indicating arboreal habits of *Archaeopteryx*. Science 259:790-793.

Feduccia, A., L. D. Martin, and S. Tarsitano. 2007. Archaeopteryx 2007: Quo vadis? Auk 124:373-380.

Fowler, D. W., E. A. Freedman, and J. B. Scannella. 2009. Predatory Functional Morphology in Raptors: Interdigital Variation in Talon Size Is Related to Prey Restraint and Immobilisation Technique. PLoS ONE 4(11): e7999. doi:10.1371/journal.pone.0007999

Fowler D.W., H. N. Woodward, E. A. Freedman, P. L. Larson, and J. R. Horner. 2011. Reanalysis of “*Raptorex kriegsteini*”: a juvenile tyrannosaurid dinosaur from Mongolia. PLoS ONE 6(6): e21376. doi:10.1371/journal.pone.0021376

Gaston, R., M. Lockley, S. G. Lucas, and A. P. Hunt. 2003. *Grallator*-Dominated Fossil Footprint Assemblages and Associated Enigmatic Footprints from the Chinle Group (Upper Triassic), Gateway Area, Colorado. Ichnos 10:153-163.

Gatesy, S. M., K. M. Middleton, F. A. Jenkins, Jr., and N. H. Shubin. 1999. Three-dimensional preservation of foot movements in Triassic theropod dinosaurs. Nature 399:141-144.

Glen, C. L., and M. B. Bennett. 2007. Foraging modes of Mesozoic birds and non-avian theropods. Current Biology 17:R911-R912.

Haffner, M. 1999. A tendon-locking mechanism in two climbing rodents, *Muscardinus avellanarius* and *Micromys minutus* (Mammalia, Rodentia), Journal of Morphology 229:219-227.

Hallager, S. 2004. North American Regional Studbook for the red-legged seriema. Smithsonian National Zoological Park, Washington DC, 49 pp.

Holtz Jr., T. R., D. L. Brinkman, and C. L. Chandler. 1998. Denticle morphometrics and a possibly omnivorous feeding habit for the theropod dinosaur *Troodon*. Gaia 15:159-166.

Kobayashi, Y., and R. Barsbold. 2005. Reexamination of a primitive ornithomimosaur, *Garudimimus brevipes* Barsbold, 1981 (Dinosauria: Theropoda), from the Late Cretaceous of Mongolia. Canadian Journal of Earth Sciences 42:1501-1521.

Manning, P. L., D. Payne, J. Pennicott, P. M. Barrett, and R. A. Ennos. 2006. Dinosaur killer claws or climbing crampons? Biology Letters 22:110-112.

Manning, P. L., L. Margetts, M. R. Johnson, P. J. Withers, W. I. Sellers, P. L. Falkingham, P. M. Mummery, P. M. Barrett, and D. R. Raymont. 2009. Biomechanics of dromaeosaurid dinosaur claws: application of x-ray microtomography, nanoindentation, and finite element analysis. The Anatomical Record 292:1397-1405.

Mayr, G., B. Pohl, and D. S. Peters. 2005. A well-preserved *Archaeopteryx* specimen with theropod features. Science 310:1483-1486.

Mayr, G., B. Pohl, S. Hartman, and D. S. Peters. 2007. The tenth skeletal specimen of *Archaeopteryx*. Zoological Journal of the Linnean Society 149:97-116.

Middleton, K. M. 2001. The Morphological Basis of Hallucal Orientation in Extant Birds. Journal of Morphology 250:51-60.

Miranda-Ribeiro. A. de. 1937. Nomes Vulgares da Seriema (Commonly used names of the Seriema bird). Revista do Museu Paulista 23:35-90.

Naish, D., Dyke, G., Cau, A., Escuillié, F. and Godefroit, P. (2011) A gigantic bird from the Upper Cretaceous of Central Asia. Biology Letters doi: 10.1098/rsbl.2011.0683

Norell, M. A., and P. J. Makovicky. 1997. Important features of the dromaeosaur skeleton: Information from a new specimen. American Museum Novitates 3215:1-28.

Norell, M. A., and P. J. Makovicky. 1999. Important features of the dromaeosaur skeleton. II. Information from newly collected specimens of *Velociraptor* *mongoliensis*. American Museum Novitates 3282:1-45.

Ostrom, J. H. 1969. Osteology of *Deinonychus* *antirrhopus*, an unusual theropod from the Lower Cretaceous of Montana. Bulletin of the Peabody Museum of Natural History 30:1-165.

Ostrom, J. H. 1976. On a new specimen of the Lower Cretaceous theropod dinosaur *Deinonychus* *antirrhopus*. Breviora 439:1-21.

Padian, K., and J. R. Horner. 2002. Typology versus transformation in the origin of birds. Trends in Ecology and Evolution 17:120-124.

Peters, D. S. and E. Görgner. 1992. A comparative study on the claws of *Archaeopteryx*; pp. 29-37 in K. E. Campbell (ed), Papers in avian paleontology Honoring Pierce Brodkorb. Science Series Natural History, Museum of Los Angeles County, Los Angeles.

Pike, A. V. L., and D. P. Maitland. 2004. Scaling of bird claws. Journal of Zoology 262:73-81.

Quinn, T. H., and J. J. Baumel. 1990. The digital tendon locking mechanism of the avian foot (Aves). Zoomorphology 109:281-293.

Redford, K.H., and G. Peters. 1986. Notes on the Biology and Song of the Red-legged Seriema (*Cariama cristata*). Journal of Field Ornithology 57:261-269.

Rozhdestvensky, A. K. 1965. Vosractnay ismenchivosty i nekotorie voprosi sistematiki dinosavrov Asii [Growth changes in Asian dinosaurs and some problems of their taxonomy]. Paleontologicheskiy Zhurnal 3: 95-109.

Senter, P. 2007. A new look at the phylogeny of Coelurosauria (Dinosauria: Theropoda) Journal of Systematic Palaeontology 5:429-463.

Sereno, P. C. 2001. Alvarezsaurids: Birds or ornithomimosaurs?; pp. 69-98 in J. A. Gauthier, and L. F Gall (eds), New Perspectives on the Origin and Early Evolution of Birds: Proceedings of the International Conference in Honor of John H. Ostrom. Peabody Museum of Natural History, New Haven, Connecticut.

Sick, H. 1993. Birds in Brazil. Princeton NJ: Princeton University Press. 932 pp.

Simmons, N.B., and T.H. Quinn. 1994. Evolution of the digital tendon locking mechanism in bats and dermopterans: a phylogenetic perspective. Journal of Mammal Evolution 2:231-254.

Tsuihiji, T., M. Watabe, K. Tsogtbaatar, T. Tsubamoto, R. Barsbold, S. Suzuki,, A. H. Lee, R. C. Ridgely, Y. Kawahara, and L. M. Witmer. 2011. Cranial osteology of a juvenile specimen of *Tarbosaurus bataar* from the Nemegt Formation (Upper Cretaceous) of Bugin Tsav, Mongolia. Journal of Vertebrate Paleontology 31: 497-517.

Turner, A. H., S. J. Nesbitt, and M. A. Norell. 2009. A large alvarezsaurid from the Late Cretaceous of Mongolia. American Museum Novitates 3648:1-14.

Wang, X., Z. Zhou, H. He, F. Jin, Y. Wang, J. Zhang, Y. Wang, X. Xu, and F. Zhang. 2005. Stratigraphy and age of the Daohugou Bed in Ningcheng, Inner Mongolia. Chinese Science Bulletin 50: 2369-2376.

Ward, A. B., P. D. Weigl, and R. M. Conroy. 2002. Functional morphology of raptor hindlimbs: implications for resource partitioning. Auk 119:1052-1063.

Xu, X., M. A. Norell, X.-L. Wang, P. J. Makovicky, and X.-C. Wu. 2002. A basal troodontid from the Early Cretaceous of China. Nature 415:780-784.

Xu, X., Ma, Q.-Y. and Hu, D.-Y. (2010) Pre-*Archaeopteryx* coelurosaurian dinosaurs and their implications for understanding avian origins. Chinese Science Bulletin 55:3971–3977.

Xu, X., You, H., Du, K. and Han, F. (2011) An *Archaeopteryx*-like theropod from China and the origin of Avialae. Nature 475, 465-470.

Zanno L. E. 2010. Osteology of *Falcarius utahensis* (Dinosauria: Theropoda): characterizing the anatomy of basal therizinosaurs. Zoological Journal of the Linnean Society 158:196-230.

Zanno, L. E., and P. J. Makovicky. 2011. Herbivorous ecomorphology and specialization patterns in theropod dinosaur evolution. Proceedings of the National Academy of Sciences 108:232-237.

Zhang, F., Z. Zhou, X. Xu, and X. Wang. 2002. A juvenile coelurosaurian theropod from China indicates arboreal habits. Naturwissenschaften 89:394–398.

Zhang, F., Z. Zhou, X. Xu, X. Wang, and C. Sullivan. A bizarre Jurassic maniraptoran from China with elongate ribbon-like feathers. Nature 455:1105-1108.
